# Comparative efficacy of traditional Chinese medicine qigong exercise on motor and non-motor outcomes in Parkinson's disease: a network meta-analysis

**DOI:** 10.3389/fneur.2026.1836123

**Published:** 2026-07-15

**Authors:** Shaoyue Xu, Yaohuan Sun, Wei Chen

**Affiliations:** School of Medicine, Hunan Normal University, Changsha, China

**Keywords:** baduanjin, liuzijue, network meta-analysis, Parkinson's disease, tai chi, traditional qigong, wuqinxi, yijinjing

## Abstract

**Background:**

This study employed a network meta-analysis (NMA) to compare the intervention effects of different TCMQE practices on primary outcome measures in PD patients and to rank their efficacy, aiming to provide guidance for clinical practice.

**Method:**

A systematic search was conducted in PubMed, Embase, Cochrane Library, Web of Science, China National Knowledge Infrastructure (CNKI), Wanfang Database, and VIP Database to identify randomized controlled trials (RCTs) evaluating traditional Chinese Qigong (QG) exercise interventions for Parkinson's disease patients. The search covered the inception of each database up to April 29, 2026. A Bayesian network meta-analysis was conducted using R software to compare the relative effects of different interventions on primary outcome measures and rank them. Two researchers independently performed literature screening and data extraction. The risk of bias in included studies was assessed using the ROB 2.0 tool.

**Results:**

A total of 59 RCTs (3,743 patients) were included. Compared with conventional treatment, Baduanjin (BDJ), QG, and Tai Chi (TC) were associated with improvements in motor symptoms (UPDRS-III). BDJ, TC, and WQX improved balance (BBS), while BDJ, liuzijue (LZJ), QG, TC, and wuqinxi (WQX) showed benefits in gait performance (TUGT). WQX and Yijingjin (YJJ) were associated with improvements in quality of life (PDQ-39), and TC and BDJ with reductions in depressive symptoms (HAMD).

**Conclusion:**

This network meta-analysis suggests that different traditional QG interventions may be associated with improvements in motor and non-motor symptoms in Parkinson's disease. However, most pairwise comparisons showed overlapping confidence intervals, and no statistically significant differences were observed between most QG interventions.

**Systematic review registration:**

https://www.crd.york.ac.uk/prospero/, identifier: CRD420251243598.

## Background

Parkinson's disease is the second most common neurodegenerative disorder globally and has emerged as the fastest-growing age-related neurological condition in recent years (1, 2). According to the Global Burden of Disease (GBD) study, approximately 11.8 million people worldwide lived with Parkinson's disease in 2021, representing an increase of over 150% since 1990 (3). This rapid growth is primarily attributed to population aging, increased life expectancy, and potential environmental exposures associated with industrialization—a phenomenon later termed the “Parkinson's epidemic”(4, 5). Age is the primary risk factor for Parkinson's disease, and the incidence rate among men is approximately 1.5 times higher than that among women (6, 7). The prevalence of the disease is particularly high in China, with a prevalence rate of approximately 1.37% among people aged 60 and older; China also has the largest number of people with Parkinson's disease in the world (8). Projections indicate that without effective disease-modifying therapies, the global burden of Parkinson's disease will continue to rise significantly by 2,040, urgently requiring optimized management strategies and enhanced targeted research (4, 9). Current clinical management primarily relies on dopaminergic medications like levodopa and Deep Brain Stimulation (DBS). While these interventions effectively alleviate core motor symptoms including bradykinesia and tremor, they cannot halt disease progression (6). Furthermore, long-term medication has limited efficacy for postural instability, gait rigidity, and non-motor symptoms like sleep disorders (10). Against this backdrop, regular physical exercise has been recognized as a vital non-pharmacological adjunct therapy. Robust medical evidence now demonstrates that consistent exercise not only markedly improves motor function in Parkinson's patients but also alleviates emotional issues such as depression and anxiety, along with sleep disturbances, thereby enhancing overall quality of life (11).

Among various adjunctive treatment strategies, traditional Chinese qigong (QG) exercises—as a mind-body integrated intervention—emphasize the synergistic effects of regulating the body, breath, and mind; its unique mind-body integration model is gradually attracting widespread attention in both clinical and research circles (12). Current research suggests that traditional QG has potential value in improving both motor and non-motor symptoms in patients with Parkinson's disease (13). However, differences among various QG practices in terms of movement structure, training intensity, and functional focus may result in inconsistent effects on specific clinical outcomes (14). From a theoretical perspective, traditional Chinese exercise modalities such as TC, BDJ, YJJ, LZJ, and WQX are all rooted in the traditional mind–body exercise philosophy, which emphasizes the integrated regulation of physical movement, breathing control, and mental focus. These practices share common principles of slow coordinated movements, postural control, and neuromuscular engagement, which provide a conceptual basis for their inclusion within a unified framework of “traditional Chinese exercise.” Previous studies have primarily focused on the overall intervention effects of traditional QG; however, there has been a lack of systematic comparisons regarding the relative advantages of different QG practices in terms of specific outcomes such as balance, motor function, mobility, and quality of life. Therefore, identifying the distinctive intervention effects of different QG practices is of great significance for optimizing individualized exercise rehabilitation programs for patients with Parkinson's disease.

Existing randomized controlled trials suggest that traditional QG may help improve relevant clinical outcomes in patients with Parkinson's disease compared to non-active controls. However, evidence for direct comparisons between different QG practices remains insufficient. At the same time, previous studies have primarily evaluated single practices or combined multiple intervention methods in their analyses, thereby limiting a systematic understanding of the relative efficacy of different practices. Therefore, this study employs a network meta-analysis to integrate direct and indirect evidence, comparing the intervention effects of different traditional QG practices on motor and non-motor outcomes in patients with Parkinson's disease, and ranking their relative efficacy to provide a basis for clinical decision-making and the optimization of rehabilitation programs.

## Methodology

This systematic review and meta-analysis will strictly adhere to the PRISMA (Preferred Reporting Items for Systematic Reviews and Meta-Analyses) guidelines (15) and will be registered on the Prospero platform (Registration number: CRD420251243598).

### Literature search

A systematic search was conducted in PubMed, Embase, Cochrane Library, Web of Science, CNKI, Wanfang, and VIP databases. The search period spanned from the inception of each database to April 29, 2026. Search terms included Parkinson's disease, QG, BDJ, TC, LZJ, WQX, and YJJ. The specific search strategy is detailed in Supplementary Table S1.

### Inclusion and exclusion criteria

This network meta-analysis included randomized controlled trials (RCTs) evaluating traditional QG interventions on clinical outcomes in Parkinson's disease patients. Eligible subjects were clinically diagnosed Parkinson's disease patients aged ≥18 years, regardless of gender, disease duration, severity, or concomitant use of antiparkinsonian medications. Traditional QG interventions included, but were not limited to, BDJ, WQX, YJJ, TC, QG, and LZJ Included studies required clear descriptions of the QG intervention protocol, including QG type, training frequency, single session duration, and total intervention period. Control groups could receive standard care, sham intervention or placebo intervention, waitlist control, or other non-QG interventions. Studies must report at least one Parkinson's disease-related outcome measure, including UPDRS-III, BBS, TUGT, PDQ-39, or HAMD, and provide complete data suitable for effect size calculations.

This study excludes retrospective studies, cohort studies, case-control studies, and other similar designs; studies where subjects were not clearly diagnosed with Parkinson's disease; studies with inadequate descriptions of QG interventions, non-comparable intervention and control groups, missing outcome data, serious methodological flaws, or duplicate publications.

### Data extraction

Two authors independently screened studies based on inclusion and exclusion criteria using EndNote software. Data from the final included studies were extracted using Excel. Disputes during screening were resolved through discussion or third-party adjudication. Extracted data comprised study characteristics (first author, publication year), population characteristics (sample size, gender, age), interventions, and outcomes.

### Risk of bias

The risk of bias of included randomized controlled trials was assessed independently by two reviewers using the Cochrane Risk of Bias 2.0 (ROB 2) tool (16). The ROB 2 tool evaluates bias across five standard domains: (1) bias arising from the randomization process, (2) bias due to deviations from intended interventions, (3) bias due to missing outcome data, (4) bias in measurement of the outcome, and (5) bias in selection of the reported result. Each domain was judged as “low risk,” “some concerns,” or “high risk” according to the ROB 2 signaling questions. Discrepancies between reviewers were resolved through discussion or consultation with a third reviewer. An overall risk-of-bias judgment was then derived from the domain-level assessments following ROB 2 guidance.

### Statistical analysis

A Bayesian network meta-analysis (17) was conducted to compare the relative efficacy of different interventions for Parkinson's disease. The primary outcomes included UPDRS-III, BBS, TUGT, PDQ-39, and HAMD scores. A network plot was first constructed, in which each intervention was treated as a node and direct comparisons between interventions were represented as edges. A Bayesian hierarchical random-effects model was applied to account for between-study variability. Treatment effects for continuous outcomes were estimated using mean differences (MDs) with corresponding 95% credible intervals (CrIs). The analysis was performed within a Markov Chain Monte Carlo (MCMC) framework using the “gemtc” package in R software (version 4.0.0). Three parallel MCMC chains were run with different initial values to ensure model stability. Convergence was assessed using the Gelman–Rubin diagnostic (potential scale reduction factor, PSRF), with values approaching one indicating satisfactory convergence. Model fit and global consistency were evaluated by comparing the deviance information criterion (DIC) between consistency and inconsistency models, with smaller DIC values indicating better model fit. Where applicable, node-splitting analysis was used to assess local inconsistency between direct and indirect evidence. Statistical heterogeneity was accounted for using a random-effects model, and was additionally evaluated using the I^2^ statistic, with values greater than 50% indicating substantial heterogeneity. When heterogeneity or inconsistency was detected, sensitivity analyses were performed to explore potential sources. Results were reported as posterior mean estimates with 95% credible intervals, along with treatment ranking probabilities derived from the surface under the cumulative ranking curve (SUCRA), providing a probabilistic ranking of interventions.

### Assessment of evidence quality

Following the completion of the Bayesian network meta-analysis, this study further applied the GRADE approach to grade the quality of evidence for key comparisons of primary outcomes. The assessment included risk of bias, inconsistency, indirectness, imprecision, and publication bias, and the evidence quality was classified into four levels: high, moderate, low, and very low. The quality of evidence for major significant comparisons was comprehensively assessed by integrating effect estimates and their 95% confidence intervals, results of bias risk assessments, results of consistency tests, and results of publication bias assessments.

## Results

### Literature search results

**Figure 1:**
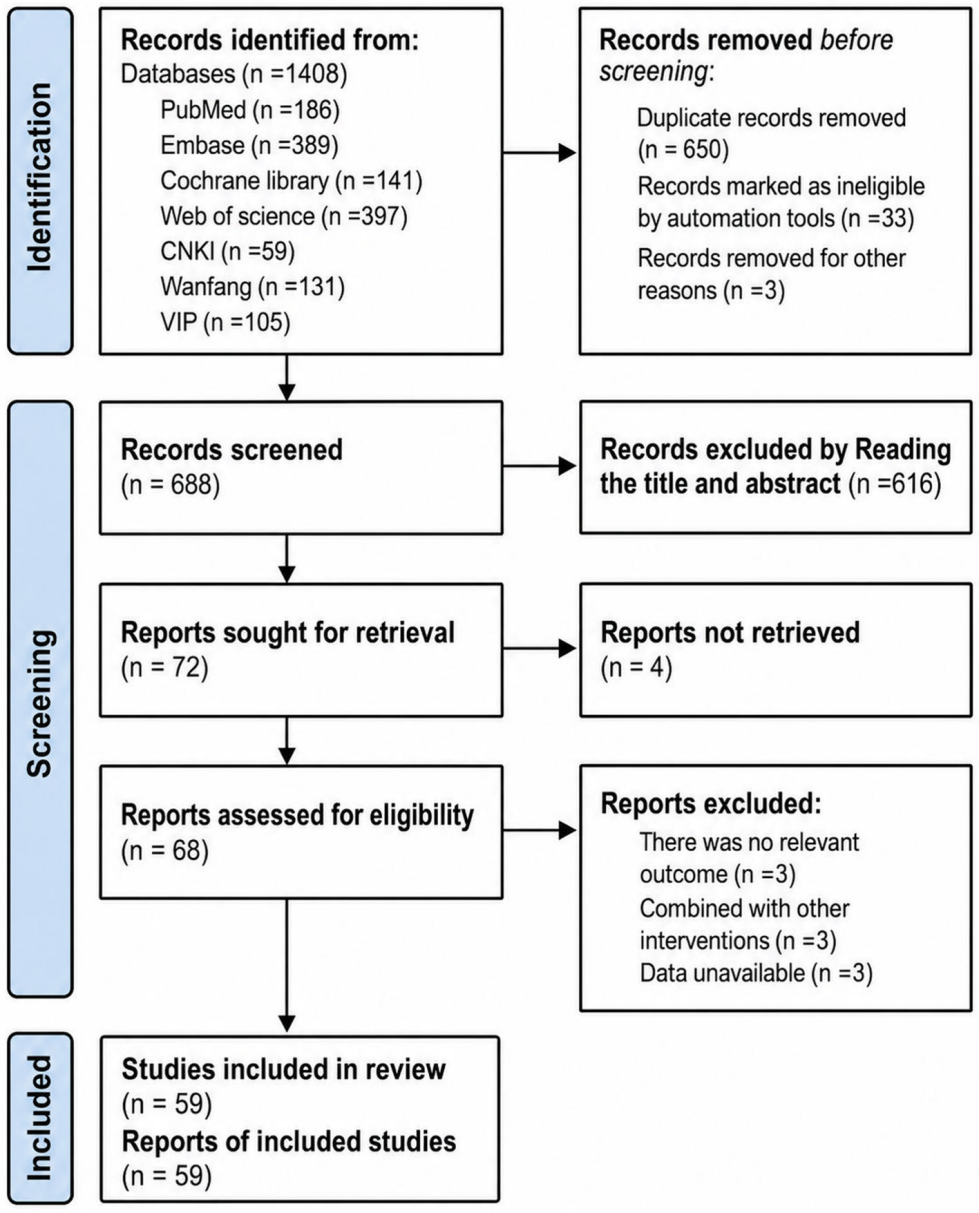
Flow chart of literature search.

As shown in Figure 1, this study retrieved a total of 1,408 articles from the following sources: PubMed (*n* = 186), Embase (*n* = 389), Cochrane Library (*n* = 141), Web of Science (*n* = 397), CNKI (*n* = 59), Wanfang (*n* = 131), and VIP (*n* = 105). After screening titles and abstracts, 616 articles were excluded. Following full-text review, 9 articles were excluded, resulting in the final inclusion of 59 articles.

### Inclusion criteria for basic characteristics

This study included 59 randomized controlled trials involving 3,743 patients and 6 interventions: QG; BDJ; TC; WQX; YJJ; and LZJ. Detailed baseline characteristics are provided in Supplementary Table S2.

### Risk of bias

Risk of bias assessments for the 59 included randomized controlled trials (RCTs) are detailed in Supplementary Figures S1 and S2. Results indicate that most studies exhibited low risk for “incomplete outcome data” and “selective reporting,” suggesting generally complete outcome data and adequate reporting. However, participant and investigator blinding represented a major source of bias, with approximately half of the studies demonstrating high risk. Concurrently, random sequence generation and allocation concealment were frequently rated as “risk of bias unclear” due to insufficient reporting of methodological details. Risks for outcome assessment blinding primarily ranged between low risk and unclear risk. Overall, methodological limitations in the included studies primarily centered on inadequate implementation of blinding and insufficient reporting of randomization procedures.

The detailed GRADE Summary of Findings for all primary outcome–intervention comparisons are presented in Supplementary Table S10. Overall, the certainty of evidence ranged from moderate to low, with no high-certainty evidence identified across outcomes. For UPDRS-III, BDJ and TC vs. control were rated as moderate-certainty evidence, while QG vs. control was rated as low certainty due to additional downgrading for imprecision. For BBS, both BDJ and TC showed moderate-certainty evidence, with downgrading mainly attributed to risk of bias. For TUGT and HAMD, the evidence was generally of low certainty, primarily due to risk of bias and imprecision. For PDQ-39, YJJ vs. control was rated as moderate certainty, downgraded mainly due to risk of bias.

### Paired meta-analysis

Results of the paired meta-analysis are presented in Supplementary Table S9. All effect estimates were standardized to the direction of “intervention vs. control,” with negative mean difference (MD) values indicating lower scores in the intervention group compared with the control group, corresponding to improved clinical outcomes for all outcomes.

Regarding UPDRS-III scores, BDJ, QG, TC, WQX, YJJ, and LZJ all showed improvements compared with conventional treatment. Specifically, BDJ vs. Control [MD = −5.33, 95% CrI (2.09, 8.52)], QG vs. Control [MD = −4.03, 95% CrI (−7.77, −0.24)], TC vs. Control [MD = −3.57, 95% CrI (−5.29, −1.85)], WQX vs. Control [MD = −2.82, 95% CrI (−5.88, 0.22)], YJJ vs. Control [MD = −2.28, 95% CrI (−9.15, 4.67)], and LZJ vs. Control [MD = −1.55, 95% CrI (−7.41, 4.34)].

For BBS outcomes, TC vs. Control [MD = 3.98, 95% CrI (2.92, 5.02)], BDJ vs. Control [MD = 3.71, 95% CrI (1.81, 5.62)], WQX vs. Control [MD = 2.94, 95% CrI (0.85, 5.05)], and QG vs. Control [MD = 1.12, 95% CrI (−3.72, 5.94)] were reported. Compared with UPDRS-III and TUGT, higher scores in BBS indicate better balance function.

For TUGT, BDJ vs. Control [MD = −3.59, 95% CrI (0.53, 7.04)], LZJ vs. Control [MD = −6.80, 95% CrI (−11.69, −1.80)], WQX vs. Control [MD = −3.51, 95% CrI (−5.71, −1.46)], QG vs. Control [MD = −3.02, 95% CrI (−6.18, −0.05)], and TC vs. Control [MD = −1.65, 95% CrI (−2.76, −0.59)] were observed, indicating improved gait performance with lower TUGT scores.

For PDQ-39, YJJ vs. Control [MD = −18.14, 95% CrI (−24.4, −11.7)], BDJ vs. Control [MD = −12.02, 95% CrI (−24.73, 0.78)], WQX vs. Control [MD = −5.45, 95% CrI (−8.58, −2.44)], TC vs. Control [MD = −3.38, 95% CrI (−7.83, 1.03)], LZJ vs. Control [MD = −1.87, 95% CrI (−9.76, 5.90)], and QG vs. Control [MD = −7.97, 95% CrI (−22.84, 7.16)] were reported.

For HAMD scores, TC vs. Control [MD = −5.20, 95% CrI (−8.92, −1.29)], BDJ vs. Control [MD = −3.88, 95% CrI (−7.58, −0.21)], WQX vs. Control [MD = −2.41, 95% CrI (−7.12, 2.30)], and LZJ vs. Control [MD = −0.60, 95% CrI (−9.43, 8.27)] were observed.

### Consistency modeling results

This study employed a random-effects model to compare the fit of consistent and inconsistent models. The absolute values of DIC differences between the two models for each outcome were all < 5, indicating comparable model fit between consistent and inconsistent models. No significant conflicts were identified between direct and indirect evidence within the network, demonstrating overall good consistency (Supplementary Table S3). Furthermore, statistical heterogeneity was generally low across outcomes: I^2^ values for UPDRS-III, BBS, and PDQ-39 were all 0%, indicating consistent results across studies. I^2^ values for TUGT and HAMD were 5 and 3%, respectively, also reflecting low levels of heterogeneity and minimal inter-study variation. Nevertheless, differences in intervention duration, training frequency, and sample size persist across studies, and these factors may influence the interpretation of results in clinical practice.

### UPDRS-III

**Figure 2:**
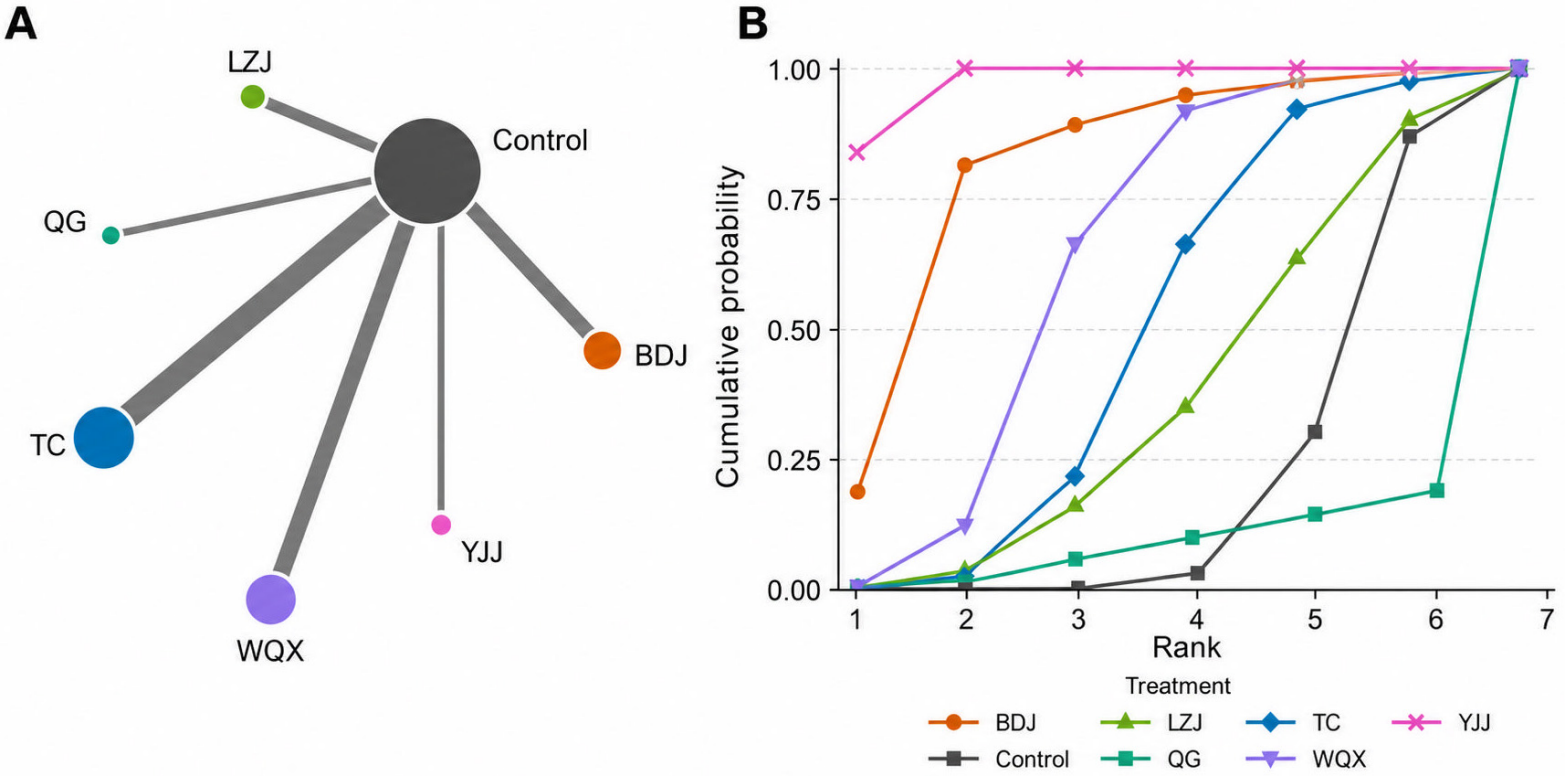
Results of network meta-analysis of UPDRS-III: **(A)** network plot; **(B)** cumulative ranking probability plot.

A total of 40 studies reporting UPDRS-III scores were included. Among these, six QG methods—TC, QG, BDJ, WQX, LZJ, and YJJ—were directly compared with conventional treatment in the network diagram (Figure 2A). According to the ranking table (Supplementary Table S4), compared with conventional treatment, BDJ [MD = −5.33, 95% CrI (−8.47, −2.17)], QG [MD = −4.18, 95% CrI (−7.49, 0.86)], and TC [MD = −3.54, 95% CrI (−5.19, −1.87)] improved motor function in Parkinson's patients. No significant differences existed among the QG methods. As indicated by the Surface Under the Cumulative Ranking Curve (SUCRA) (Figure 2B and Table 1), The ranked probabilities of improving motor function for each intervention were: BDJ (84.7%), QG (69.4%), TC (59.6%), WQX (48.2%), YJJ (43.6%), LZJ (34.8%).

**Table 1:** Results of SUCRA ranking for UPDRS-III, BBS, PDQ-39, TUGT, and HAMD.

Treatment	UPDRS-III (%)	BBS (%)	PDQ-39 (%)	TUGT (%)	HAMD (%)
BDJ	84.7	74.1	79.6	62.6	68.6
Control	9.71	8.1	20.5	0.8	15
LZJ	34.8	NR	36.7	92.5	32.7
QG	69.4	29.8	7.8	53.5	NR
TC	59.6	82.2	46.7	27.7	84.4
WQX	48.2	55.8	61.9	62.8	49.3
YJJ	43.6	NR	96.7	NR	NR

### BBS

**Figure 3:**
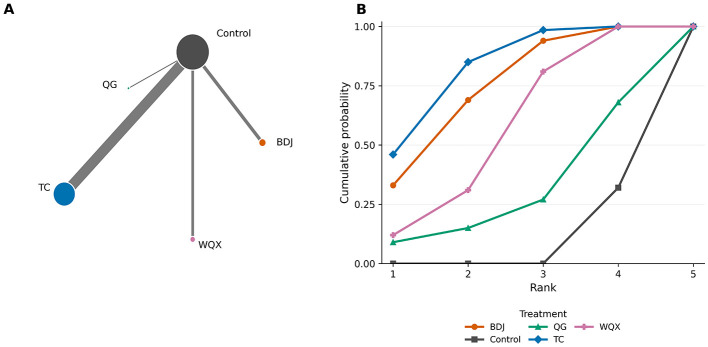
Results of network meta-analysis of BBS: **(A)** network plot; **(B)** cumulative ranking probability plot.

A total of 33 studies reporting BBS score data were included. The network diagram indicates direct comparative evidence exists between TC, BDJ, WQX, and QG interventions and standard care (Figure 3A). According to the ranking table (Supplementary Table S5), compared with conventional treatment, BDJ [MD = 3.72, 95%CrI (1.82, 5.62)], TC [MD = 3.98, 95%CrI (2.91, 5.02)], WQX [MD = 2.94, 95%CrI (0.85, 5.04)] improved balance in Parkinson's patients. No significant differences existed among QG interventions. SUCRA ranking results (Figure 3B and Table 1) indicated the following probabilities for each intervention improving balance function: TC (82.2%), BDJ (74.1%), WQX (55.8%), and QG (29.8%).

### PDQ-39

**Figure 4:**
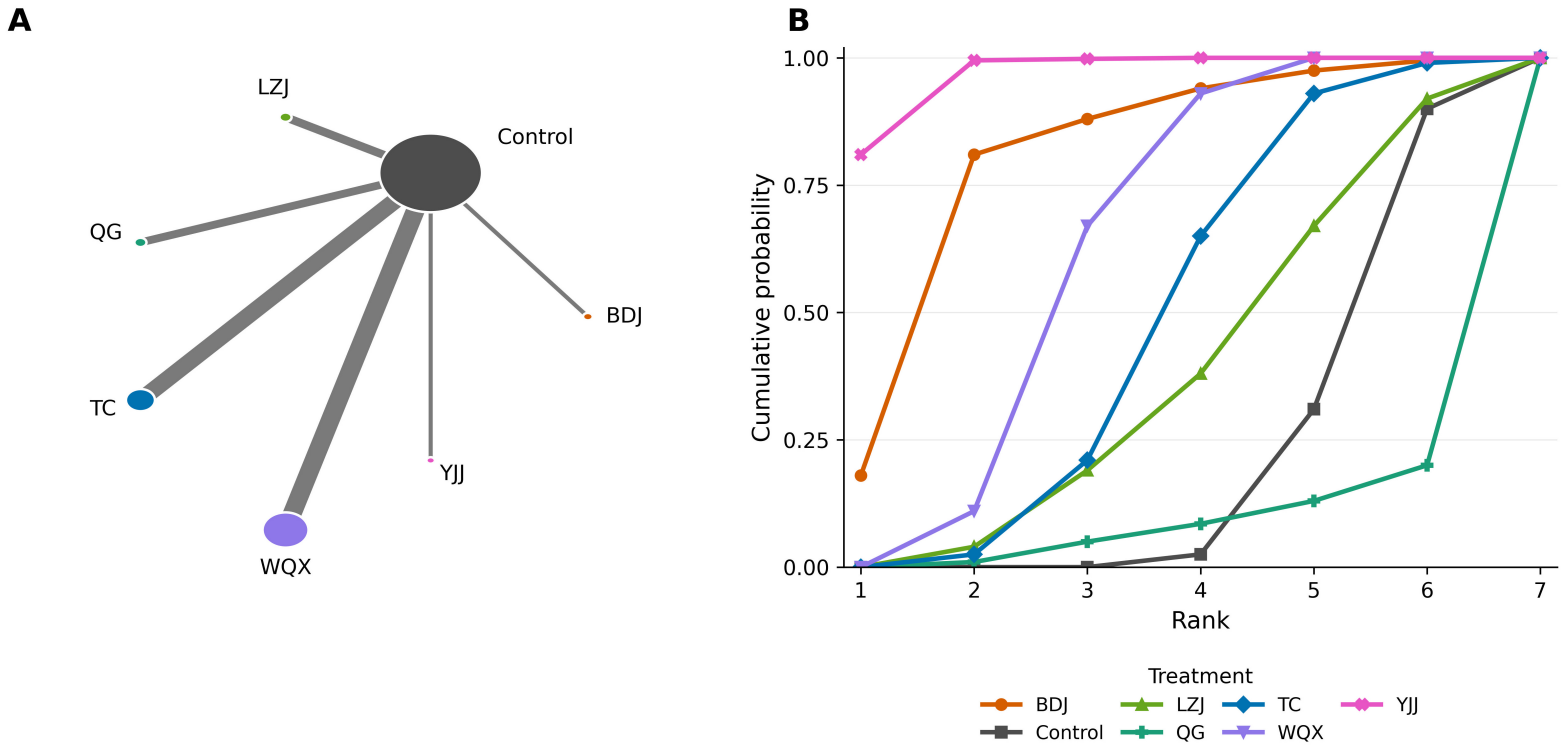
Results of network meta-analysis of PDQ-39: **(A)** network plot; **(B)** cumulative ranking probability plot.

A total of 16 studies reporting PDQ-39 scores were included. Among these, six QG methods—YJJ, BDJ, WQX, TC, LZJ, and QG—were directly compared with conventional therapy in the network diagram (Figure 4A). According to the ranking table (Supplementary Table S7), compared with conventional therapy, WQX [MD = −5.44, 95%CrI (−8.6, −2.45)], YJJ [MD = −18.14, 95%CrI (−24.49, −11.76)] improved quality of life in Parkinson's patients. YJJ demonstrated a greater degree of quality-of-life improvement than WQX [MD = −12.71, 95%CrI (−19.68, −5.48)]. SUCRA ranking results (Figure 4B and Table 1) indicate the probability order of quality-of-life improvement among interventions: YJJ (96.7%), BDJ (79.6%), WQX (61.9%), TC (46.7%), LZJ (36.7%), and QG (7.8%).

### TUGT

**Figure 5:**
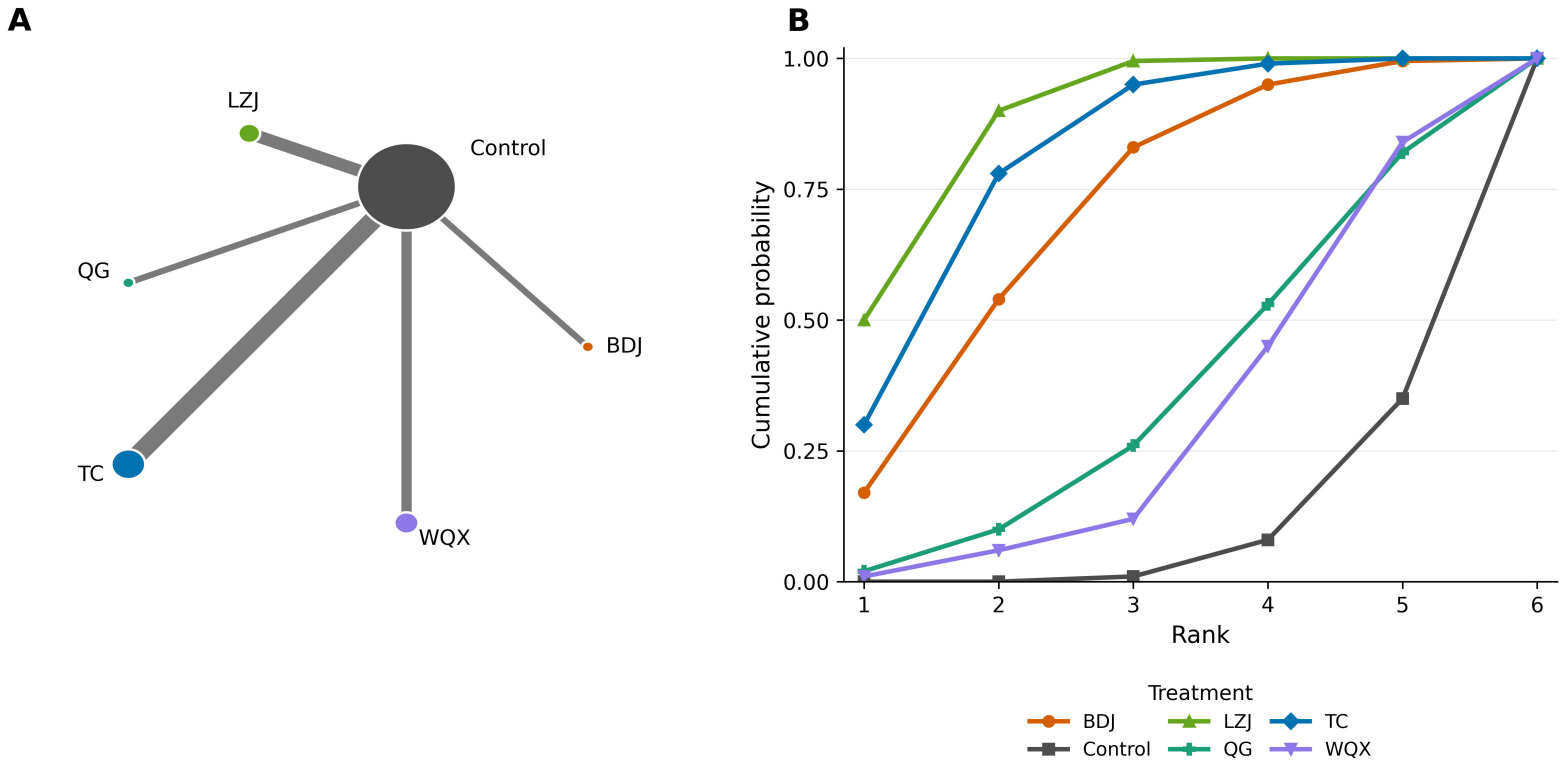
Results of network meta-analysis of TUGT: **(A)** network plot; **(B)** cumulative ranking probability plot.

A total of 21 studies reporting TUGT scores were included. Among these, five QG methods—TC, WQX, QG, BDJ, and LZJ—were directly compared with conventional treatment in the network diagram (Figure 5A). According to the ranking table (Supplementary Table S6), compared with conventional treatment, BDJ [MD = −3.56, 95%CrI (−7.06, −0.53)], LZJ [MD = −6.8, 95%CrI (−11.74, −1.87)], QG [MD = −3.01, 95%CrI (−6.12, −0.05)], TC [MD = −1.65, 95%CrI (−2.75, −0.61)], WQX[MD = −3.49, 95%CrI(−5.67, −1.44)] can improve gait in Parkinson's patients, and LZJ demonstrates greater gait improvement than TC [MD = −5.14, 95%CrI (−10.17, −0.1)]. SUCRA ranking results (Figure 5B and Table 1) indicate the ranked probabilities of improving gait function for each intervention were: LZJ (92.5%), WQX (62.8%), BDJ (62.6%), QG (53.5%), and TC (27.7%).

### HAMD

**Figure 6:**
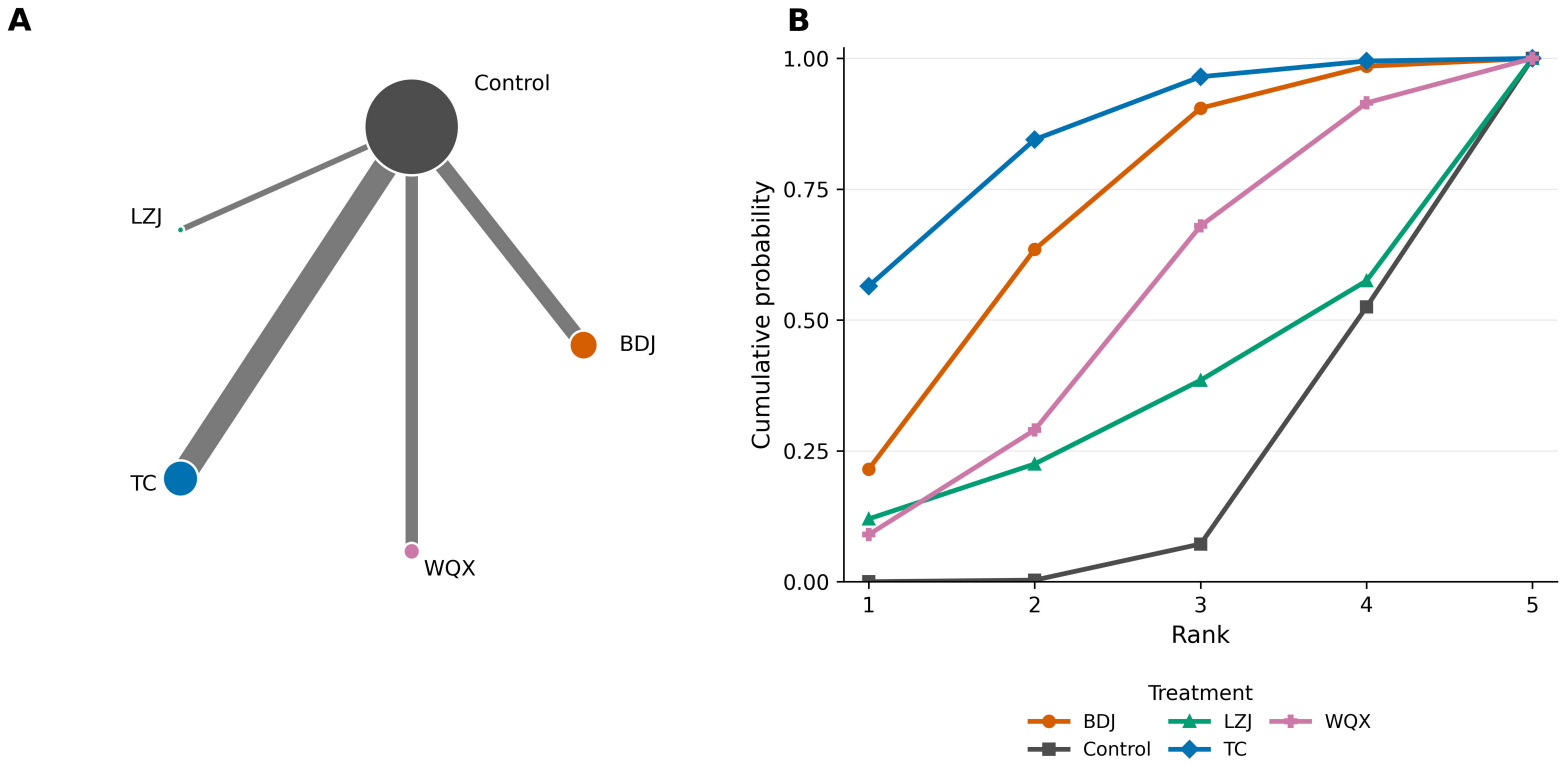
Results of network meta-analysis of HAMD: **(A)** network plot; **(B)** cumulative ranking probability plot.

Thirteen studies reporting HAMD scores were included. Among these, four QG methods—TC, BDJ, WQX, and LZJ—were directly compared with conventional treatment in the network diagram (Figure 6A). According to the ranking table (Supplementary Table S8), compared with conventional treatment, TC [MD = −5.19, 95%CrI (−8.93, −1.36)] and BDJ [MD = −3.87, 95%CrI (−7.58, −0.21)] significantly improved depressive symptoms in Parkinson's patients, with no significant differences among the QG methods. SUCRA ranking indicated that LZJ also improved depressive symptoms. BDJ [MD = −3.87, 95%CrI (−7.58, −0.21)] improved depressive symptoms in Parkinson's patients. No significant differences existed among QG methods. SUCRA ranking results (Figure 6B and Table 1) indicated the following probabilities for each intervention improving depressive symptoms: TC (84.4%), BDJ (68.6%), WQX (49.3%), and LZJ (32.7%).

### Subgroup analysis results

Subgroup analyses (Supplementary Table S11) were conducted to explore the potential effects of intervention duration, training frequency, intervention dose, and disease severity (Hoehn and Yahr stage) on treatment outcomes. For UPDRS-III scores, longer intervention duration (>12 weeks) and higher training frequency (>3 sessions per week) were associated with greater improvements compared with shorter and lower-frequency interventions. Similarly, patients with earlier disease stage (Hoehn and Yahr stage 1–2) showed greater reductions in motor symptoms compared with those in more advanced stages. For BBS outcomes, both longer duration and higher frequency interventions demonstrated superior effects on balance function, with consistent trends across subgroups. For TUGT outcomes, longer and higher-frequency interventions were associated with larger reductions in time required to complete the test, indicating improved gait performance. For PDQ-39, high-dose and longer-duration interventions showed greater improvements in quality of life compared with low-dose and short-duration interventions. For HAMD scores, patients in earlier disease stages demonstrated greater improvements in depressive symptoms, while those in advanced stages showed relatively smaller effects.

### Publication bias detection

This study employed funnel plots to detect publication bias. The results (see Supplementary Figures S3–S7) revealed relatively symmetrical funnel plots, indicating a low likelihood of publication bias. Quantitative assessment using Egger's test indicated no significant evidence of publication bias for the main outcomes, including UPDRS-III (*P* = 0.09), BBS (*P* = 0.27), TUGT (*P* = 0.16), PDQ-39 (*P* = 0.11), and HAMD (*P* = 0.73).

## Discussion

This study employed a Bayesian network meta-analysis to compare the effects of different traditional qigong interventions on multidimensional outcomes in patients with Parkinson's disease. The results showed that BDJ had a higher-ranking probability for improving motor symptoms; TC demonstrated relatively favorable ranking results for improving balance function and depressive symptoms; LZJ may have certain advantages in gait function; and YJJ showed a higher-ranking probability in terms of improvements in health-related quality of life. It should be emphasized that SUCRA primarily reflects the relative ranking probabilities of each intervention across all comparisons and does not directly represent the absolute magnitude of treatment differences or clinical certainty of relatively higher-ranking probabilities. Therefore, SUCRA results should be interpreted in conjunction with effect sizes and their 95% confidence intervals, and, when necessary, in combination with the consistency and certainty of the evidence; for comparisons where confidence intervals overlap or differences do not reach statistical significance, the relevant ranking results should still be interpreted with caution, and clear clinical priority recommendations should not be made based solely on these results. Overall, the ranking results for each outcome in this study were generally consistent with the direction of the corresponding effect sizes, suggesting a relatively stable overall trend across different studies. This may be related to the fact that all included participants were patients with Parkinson's disease, the interventions were primarily based on traditional fitness qigong systems, and most studies used relatively consistent outcome assessment tools. However, differences still exist among studies regarding intervention duration, training frequency, sample size, and control measures; these factors may influence intervention outcomes at the clinical level. Furthermore, the results from the consistency model were like those from the inconsistency model, suggesting no apparent conflicts among the networked evidence, which provides some methodological support for the robustness of this study's findings (18).

Building on this, the present study further applied the GRADE approach to assess the quality of evidence for key comparisons of primary outcomes. The results showed that the quality of evidence for each outcome was generally moderate to low, with no high-quality evidence identified. Most comparisons were downgraded primarily due to risk of bias, and some were further downgraded due to imprecision, suggesting that the overall certainty of the existing evidence remains limited. Therefore, although some traditional QG interventions demonstrated certain advantages in several outcomes, conclusions regarding the relative efficacy of different interventions should be drawn with caution. Conclusions should not be based solely on ranking results but should be interpreted comprehensively considering effect sizes and the quality of evidence.

In the UPDRS-III scoring outcomes, BDJ demonstrated a higher probability of improving motor symptoms in Parkinson's disease patients. This may be closely related to the exercise's movement structure, which aligns well with the rehabilitation needs for PD motor impairments. The core motor symptoms of PD primarily manifest as bradykinesia, resting tremor, and muscle rigidity, with their pathological basis attributed to dysfunction in the basal ganglia-cortical motor circuitry (19). BDJ incorporates large-amplitude, slow-paced, and highly controllable limb extension movements alongside multi-directional spinal activities, exemplified by postures like “Lifting Hands to the Sky to Regulate the Triple Burner” and “Shaking the Head and Wagging the Tail to Clear Heart Fire.” These movements systematically expand joint range of motion, correct forward-leaning posture, and enhance muscle strength, thereby effectively alleviating muscle rigidity (20). Furthermore, BDJ organically integrates deep breathing with mental regulation into its movements. Research by Xiao et al. suggests this characteristic may reduce stress-related tremors by regulating autonomic nervous system balance, enhancing parasympathetic tone, and inhibiting excessive sympathetic activation (21). Additionally, BDJ's sustained training in dynamic center-of-gravity shifts and postural control activates proprioceptive afferent pathways, aiding in the reconstruction of impaired sensorimotor coordination in patients (22). Regarding neuroplasticity mechanisms, recent functional Magnetic Resonance Imaging (fMRI) studies indicate that regular BDJ practice enhances connectivity between the primary motor cortex and supplementary motor areas (23). Compared to other QG practices, BDJ emphasizes bilateral symmetrical movements and postural stability control in its choreography. Bilateral coordination training has been demonstrated to uniquely improve gait symmetry and overall motor performance in PD patients (24), which may be a key reason for BDJ's relative advantage in UPDRS-III composite scores.

BBS and HAMD scoring results indicate that TC demonstrates relatively favorable outcomes in improving balance function and alleviating depressive symptoms. PD patients commonly exhibit impaired postural reflexes, leading to a markedly elevated risk of falls. The core elements of TC training involve sustained dynamic shifts in center of gravity and active adjustments to the base of support. This process continuously strengthens proprioceptive input and optimizes vestibular-somatosensory integration efficiency, thereby systematically enhancing postural stability (25). A randomized controlled trial by Li et al., published in the New England Journal of Medicine, demonstrated that compared to resistance training and stretching exercises, TC more significantly improved balance function and reduced fall incidence in PD patients. The mechanism may involve enhanced ankle joint proprioception and improved vestibular-somatosensory coordination efficiency (26), This conclusion has been supported by evidence from multiple subsequent meta-analyses (27, 28), and the present study's findings align with these results. Due to its slow movements and focused mental intent, TC is often regarded as a form of dynamic meditation. Previous studies have shown that regular TC practice can downregulate excessive activation of the hypothalamic-pituitary-adrenal (HPA) axis, reduce serum cortisol levels and concentrations of pro-inflammatory cytokines (such as IL-6), exerting antidepressant and anxiolytic effects through neuroendocrine pathways (29, 30). Furthermore, neuroimaging studies suggest that TC practice enhances activation in the dorsolateral prefrontal cortex, a brain region closely associated with emotional regulation and cognitive control. This functional improvement may further mediate the alleviation of depressive symptoms in patients (31). The synergistic interaction of these multidimensional mechanisms likely constitutes the key neurobiological basis for TC's significant effects on non-motor outcomes.

The PDQ-39 assessment results indicate that YJJ has a high probability of ranking higher in improving health-related quality of life among Parkinson's disease patients. This may be closely related to the unique isometric contraction strength training characteristics of YJJ. YJJ movements emphasize sustained force generation at the extremities and static muscle control. Forms like the “Reaching for the Stars” posture require practitioners to maintain continuous muscle tension in specific positions. This training pattern facilitates recruitment of high-threshold motor units, systematically enhancing strength and endurance in the lower limb extensor muscles (32, 33). Given that the PDQ-39 encompasses multiple dimensions including mobility, activities of daily living, emotional state, and social support, significant improvements in muscle strength can directly translate into greater independence and functional autonomy during daily activities. This, in turn, yields broad benefits in quality-of-life scores (34). Supporting prior research, Wang et al. (35) network meta-analysis indicated that traditional exercise interventions incorporating resistance or strength training components outperformed flexibility-focused programs in improving physical function among PD patients. The findings of this study on Yijin Jing corroborate the aforementioned conclusions. Furthermore, the training philosophy of “mind-body coordination” emphasized in Yijin Jing—the high integration of mental focus and physical movements—may enhance patients' self-efficacy and reduce disease-related stigma, thereby promoting their willingness to participate in social activities and increasing their frequency of social engagement (36, 37). This psychosocial improvement pathway aligns closely with changes in the social support and emotional dimensions of the PDQ-39, potentially constituting a multidimensional intervention mechanism through which Yijin Jing enhances quality of life.

TUGT results indicate that LZJ may offer certain advantages in reducing completion time and improving gait function. This may be closely related to the unique “breath-controlled vocalization training” model of LZJ. Freezing of gait (FOG) and startle difficulty are major risk factors for falls in PD patients, fundamentally stemming from impaired rhythmic generation mechanisms within the basal ganglia (38). Through the coordinated interaction of specific breathing patterns and vocalization, LZJ enhances synergistic contraction of the diaphragm and transverse abdominis muscles, elevates intra-abdominal pressure, and strengthens core stability (39). Simultaneously, rhythmic breathing and vocalization serve as endogenous auditory rhythm cues, helping patients establish stable gait rhythm output and thereby improving start-up difficulties and gait freezing (40). Research by Gilat et al. indicates that rhythmic external stimuli activate the prefrontal-cerebellar compensatory pathway, partially substituting for the impaired rhythm-generating function of the basal ganglia (41). The endogenous vocal rhythm of LZJ aligns closely with the aforementioned compensatory mechanisms in its pathway of action. Its gait-improving pattern also corresponds to the intervention mechanism of Rhythmic Auditory Stimulation (RAS) (42, 43), Clinical studies further confirm that regular LZJ practice significantly immay provide supportive evidence for step length and frequency in PD patients (42).The systematic optimization of gait parameters ultimately manifests as reduced TUGT duration. This test encompasses a series of complex functional movements—rising, straight-line walking, turning, and returning—demanding high requirements for gait initiation and dynamic balance control. Consequently, it comprehensively reflects the integrated motor benefits of LZJ intervention.

### Strengths and limitations

This study has several notable strengths. We applied relatively strict eligibility criteria and used a Bayesian network meta-analysis to combine direct and indirect evidence, which enabled simultaneous comparisons across different traditional QG interventions. In addition, multiple English and Chinese databases were searched systematically to maximize the comprehensiveness of evidence identification. We also evaluated model consistency by comparing consistency and inconsistency models, and the results suggested no major inconsistency within the network.

Several limitations of this study should be acknowledged when interpreting the findings. First, although a relatively large number of randomized controlled trials were included, the overall methodological quality of the evidence was moderate, with common concerns related to inadequate reporting of randomization procedures, allocation concealment, and blinding, which may introduce potential bias. Second, substantial clinical heterogeneity existed across studies, including variations in intervention duration, training frequency, intensity, and baseline disease severity. Although subgroup classification of traditional Qigong modalities and the use of random-effects Bayesian models were applied to mitigate these issues, residual heterogeneity cannot be fully excluded. Third, the majority of included evidence was based on comparisons between active interventions and conventional treatment, with limited direct head-to-head comparisons between different exercise modalities. This star-shaped network structure may reduce the robustness of indirect comparisons and should be considered when interpreting SUCRA-based ranking results. Finally, most included studies focused on short-term outcomes, and evidence regarding long-term effectiveness remains insufficient. In addition, differences in reporting of medication status and disease severity limited further adjustment for potential confounders.

## Conclusion

The findings of this study indicate that traditional QG interventions may provide beneficial effects on motor and non-motor outcomes in Parkinson's disease. However, most pairwise comparisons showed no statistically significant differences, with overlapping confidence intervals across interventions. A statistically significant difference was observed only between YJJ and WQX for PDQ-39 outcomes. No other significant differences were identified among the included Qigong interventions. Although SUCRA rankings suggested potential differences in probabilistic ordering, these results should be interpreted cautiously, as they do not necessarily reflect clinically meaningful or statistically significant differences.

## Data Availability

The original contributions presented in the study are included in the article/Supplementary material, further inquiries can be directed to the corresponding author.
