# Supplementary material for: Comparative efficacy of traditional Chinese medicine qigong exercise on motor and non-motor outcomes in Parkinson's disease: a network meta-analysis
Source: Front Neurol. 2026 Jul 15;17:1836123. doi: 10.3389/fneur.2026.1836123 (PMC13417681; doi:10.3389/fneur.2026.1836123)
Supplement: Supplementary file 1 [file Data_Sheet_1.docx]

Table S1 Retrieval strategy

((Parkinson Disease[MeSH Terms]) OR ((((((((((((Parkinson Disease[Title/Abstract]) OR (Idiopathic Parkinson Disease[Title/Abstract])) OR (Idiopathic Parkinson's Disease[Title/Abstract])) OR (Lewy Body Parkinson Disease[Title/Abstract])) OR (Lewy Body Parkinson's Disease[Title/Abstract])) OR (Paralysis Agitans[Title/Abstract])) OR (Parkinson Disease, Idiopathic[Title/Abstract])) OR (Parkinson's Disease[Title/Abstract])) OR (Parkinson's Disease, Idiopathic[Title/Abstract])) OR (Parkinson's Disease, Lewy Body[Title/Abstract])) OR (Primary Parkinsonism[Title/Abstract])) OR (Parkinsonism, Primary[Title/Abstract]))) AND (((Qigong[MeSH Terms]) OR (Tai Ji[MeSH Terms])) OR ((((((((((((((((((Qigong[Title/Abstract]) OR (Ch'i Kung[Title/Abstract])) OR (Qi Gong[Title/Abstract])) OR (Tai Ji[Title/Abstract])) OR (Tai-ji[Title/Abstract])) OR (Tai Chi[Title/Abstract])) OR (Chi, Tai[Title/Abstract])) OR (Tai Chi Chuan[Title/Abstract])) OR (Taiji[Title/Abstract])) OR (Taijiquan[Title/Abstract])) OR (T'ai Chi[Title/Abstract])) OR (Tai Ji Quan[Title/Abstract])) OR (Ji Quan, Tai[Title/Abstract])) OR (Quan, Tai Ji[Title/Abstract])) OR (Baduanjin[Title/Abstract])) OR (Yijinjing[Title/Abstract])) OR (Wuqinxi[Title/Abstract])) OR (Liuzijue[Title/Abstract])))

("parkinson disease"[MeSH Terms] OR ("parkinson disease"[Title/Abstract] OR "idiopathic parkinson disease"[Title/Abstract] OR "idiopathic parkinson s disease"[Title/Abstract] OR "lewy body parkinson disease"[Title/Abstract] OR "lewy body parkinson s disease"[Title/Abstract] OR "paralysis agitans"[Title/Abstract] OR "parkinson disease idiopathic"[Title/Abstract] OR "parkinson s disease"[Title/Abstract] OR "parkinson s disease idiopathic"[Title/Abstract] OR "parkinson s disease lewy body"[Title/Abstract] OR "primary parkinsonism"[Title/Abstract] OR "parkinsonism primary"[Title/Abstract])) AND ("Qigong"[MeSH Terms] OR "Tai-ji"[MeSH Terms] OR ("Qigong"[Title/Abstract] OR "ch i kung"[Title/Abstract] OR "qi gong"[Title/Abstract] OR "Tai-ji"[Title/Abstract] OR "Tai-ji"[Title/Abstract] OR "tai chi"[Title/Abstract] OR "chi tai"[Title/Abstract] OR "tai chi chuan"[Title/Abstract] OR "Taiji"[Title/Abstract] OR "Taijiquan"[Title/Abstract] OR "t ai chi"[Title/Abstract] OR "tai ji quan"[Title/Abstract] OR "ji quan tai"[Title/Abstract] OR "quan tai ji"[Title/Abstract] OR "Baduanjin"[Title/Abstract] OR "Yijinjing"[Title/Abstract] OR "Wuqinxi"[Title/Abstract] OR "Liuzijue"[Title/Abstract]))

Table S2 Include the basic characteristics of the literature and the reference

| Study | Year | Country | Sample size | Gender(M/F) | Mean age | Intervention | Outcomes |
| --- | --- | --- | --- | --- | --- | --- | --- |
| Hwa-Jin Lee | 2018 | Korea | QG:25  Control:16 | 17/24 | QG:65.8  Control:65.7 | QG  60min/day | UPDRS-Ⅲ; BBS |
| WH Wang | 2020 | China | LZJ：20  Control:21 | 23/18 | LZJ：64.23  Control:64.64 | LZJ  25min/day  5day/week  6weeks | UPDRS-Ⅲ;TUGT;PDQ-39 |
| HH Cao | 2021 | China | WQX:31  Control:31 | 41/21 | WQX:62.45  Control:62.97 | WQX  30min/day  5day/week  8weeks | PDQ-39;TUGT;BBS |
| TT Wu | 2018 | China | TC:28  Control:24 | 37/15 | TC:62.42  Control:64.66 | TC  40min/day  4day/week  16weeks | PDQ-39 |
| XH Guan | 2018 | China | TC:40  Control:40 | 44/36 | TC:69.46  Control:68.61 | TC  60min/day  4day/week  24weeks | TUGT;BBS |
| M Yang | 2025 | China | TC:32  Control:30 | 39/23 | TC:58.22  Control:59.27 | TC  60min/day  3day/week  12weeks | UPDRS-Ⅲ;BBS;TUGT |
| XH Guan | 2016 | China | TC:31  Control:31 | 33/29 | TC:70.23  Control:69.71 | TC  60min/day  4day/week  12weeks | TUGT;BBS |
| Y Zhu | 2011 | China | TC:20  Control:20 | 23/17 | TC:63.35  Control:64.83 | TC  30-45min/day  5day/week  4weeks | UPDRS-Ⅲ;BBS |
| FL Lu | 2017 | China | TC:8  Control:8 | 10/6 | TC:68.20  Control:67.75 | TC  40-60min/day  5day/week  8weeks | UPDRS-Ⅲ;BBS |
| SQ Ji | 2016 | China | TC:16  Control:16 | 17/15 | TC:56.06  Control:59.13 | TC  60min/day  12weeks | UPDRS-Ⅲ;BBS |
| JH Jiang | 2023 | China | YJJ：20  Control:20 | 21/19 | YJJ：60.60  Control:59.45 | YJJ  30min/day  5day/week  8weeks | UPDRS-Ⅲ;PDQ-39 |
| XL Liu | 2017 | China | QG：23  Control：18 | 15/26 | QG：57.2  Control：57.1 | QG  60min/day  5day/week  10weeks | TUGT;UPDRS-Ⅲ |
| MH Wang | 2023 | China | TC1:15  TC2:15  Control:15 | 24/21 | TC 1：72.07  TC 2：69.80  Control：67.13 | TC 1  40min/day  3day/week  24weeks  TC2  60min/day  3day/week  24weeks | TUGT;UPDRS-Ⅲ;BBS;HAMD |
| Q Li | 2025 | China | BDJ：50  Control：50 | 41/59 | BDJ:65.43  Control：66.41 | BDJ  30min/day  5day/week  12weeks | UPDRS-Ⅲ;BBS; HAMD |
| W Han | 2021 | China | WQX：50  Control：50 | 49/51 | WQX_MSG：68.60  Control：67.88 | WQX  40min/day  3day/week  8weeks | UPDRS-Ⅲ;BBS;PDQ-39;HAMD |
| L Zeng | 2025 | China | BDJ:31  Control：35 | 37/29 | BDJ_TCM:67.8  Control：69.4 | BDJ  20min/day  5day/week  12weeks | UPDRS-Ⅲ;BBS |
| Gloria Vergara-Diaz, MD | 2018 | America | TC:16  Control：16 | 16/16 | TC:65.7  Control：62 | TC  60min/week  24weeks | UPDRS-Ⅲ;PDQ-39;TUGT |
| Sanghee Moon | 2020 | America | LZJ:8  Control:9 | 10/7 | LZJ:66.4  Control:65.9 | LZJ  Collective  60min/week  12weeks  Home 15-20min/day  Twice/day | PDQ-39;UPDRS-Ⅲ |
| Jinse Park | 2025 | Korea | TC：29  Control：28 | 50/49 | TC：68.2  Control：68.7 | TC  60min/day  2day/week  24weeks | UPDRS-Ⅲ;TUGT |
| ZL Li | 2022 | China | WQX：20  Control：20 | 29/11 | WQX：67.57  Control：70 | WQX  90min/day  2day/week  12weeks | UPDRS-Ⅲ;TUGT;PDQ-39 |
| CL Chang | 2024 | China | TC：16  Control：13 | 18/25 | TC：66.31  Control：63.15 | TC  60min/day  2day/week  12weeks | UPDRS-Ⅲ |
| CM Xiao | 2016 | China | BDJ:48  Control：48 | 67/29 | BDJ:68.17  Control：66.52 | BDJ  45min/day  4day/week  24weeks | UPDRS-Ⅲ;BBS;TUGT |
| MJ Zhu | 2020 | China | TC:19  Control：22 | 25/16 | TC:68.53  Control：67.77 | TC  40-50min/day  3day/week  12weeks | UPDRS-Ⅲ;BBS;PDQ-39;HAMD |
| XY Li | 2022 | China | QG:15  Control:16 | 11/20 | QG:65.87  Control:63.25 | QG  60min/day  5day/week  12weeks | UPDRS-Ⅲ;TUGT |
| HM Yin | 2025 | China | LZJ:25  Control:26 | 24/27 | LZJ:58.8  Control:58.15 | LZJ  30min/day  5day/week  12weeks | UPDRS-Ⅲ;HAMD;PDQ-39 |
| ZH Wang | 2022 | China | WQX:23  SE:22  Control:25 | 28/42 | WQX:68.83  SE:67.95  Control:66.2 | WQX  90min/day  3day/week  24weeks | TUGT;PDQ-39;UPDRS-Ⅲ |
| L Meng | 2023 | China | TC:15  Control:15 | 16/14 | TC:65.6  Control:64.93 | TC  60min/day  2day/week  12weeks | UPDRS-Ⅲ;BBS |
| Q Gao | 2014 | China | TC:37  Control:39 | 50/26 | 24-FormYang-Style TC:69.54  Control:68.28 | TC  60min/day  3day/week  12weeks | UPDRS-Ⅲ;BBS;TUGT |
| Arva Khuzema | 2020 | India | TC:9  Yoga:9  Control:9 | 19/8 | TC:72H  Yoga:68.11  Control:70.89 | TC  30-40min/day  5day/week  8weeks | BBS;TUGT |
| G Li | 2022 | China | TC:32  BK:31  Control:32 | 58/37 | TC:62.7  BK:61.9  Control:61.9 | TC  60min/day  5day/week  1year | UPDRS-Ⅲ;BBS;TUGT |
| Schmidt | 2016 | America | QG:7  Control:7 | 9/5 | QG:64.5  Control:67 | QG  Collective 60min/week  6weeks  Home 15-20min/day  Twice/day | PDQ-39;UPDRS-Ⅲ |
| Schmitz-Hu¨bsch | 2006 | Germany | QG:32  Control:24 | 43/13 | QG:64  Control:63 | QG  60min/week  24weeks | UPDRS-Ⅲ |
| FZ Li | 2012 | America | TC:65  RT:65  SE:65 | 123/72 | TC:68  RT:69  SE:69 | TC  60min/day  2day/week  24weeks | UPDRS-Ⅲ;TUGT |
| Madeleine E. Hackney | 2008 | America | TC:13  Control:13 | 21/5 | TC:64.9  Control:62.6 | TC  60min/day  2day/week  13weeks | UPDRS-Ⅲ |
| Shinichi Amano | 2013 | America | Project1  TC:12  QG:9  Project2  TC:15  Control:9 | Project1:14/7  Project2:14/10 | Project1  TC:64  QG:68  Project2  TC:66  Control:66 | Project1  60min/day  2day/week  16weeks  Project2  60min/day  3day/week  16weeks | UPDRS-Ⅲ |
| KF Li | 2024 | China | BDJ:27  Control:27 | 23/31 | BDJ:65.59  Control:60,48 | BDJ  40min/day  5day/week  4weeks | UPDRS-Ⅲ;PDQ-39 |
| MY Shen | 2021 | China | WQX:15  Control:15 | 20/10 | WQX:68.67  Control:66.93 | WQX  90min/day  2day/week  12weeks | TUGT |
| Joe R Nocera | 2013 | America | TC：15  Control：6 | 11/10 | TC：66  Control：65 | TC  60min/day  3day/week  16weeks | PDQ-39 |
| CX Lu | 2024 | China | BDJ:20  Control:20 | 21/19 | BDJ:63.65  Control:64.05 | BDJ  50 min/day  4weeks | HAMD |
| XW Shi | 2021 | China | BDJ:65  Control:64 | 68/61 | BDJ:67.89  Control:67.48 | BDJ  60min/day  4day/week  8weeks | UPDRS-Ⅲ;BBS |
| CX Lu | 2024 | China | BDJ:30  Contro:30 | 34/26 | BDJ:66.70  Contro:66.57 | BDJ  60min/day  4weeks | UPDRS-Ⅲ;TUGT;BBS |
| LM He | 2024 | China | BDJ:48  Control:48 | 55/41 | BDJ:59.32  Control:61.59 | BDJ  60min/day  5day/week  8weeks | HAMD |
| H Chen | 2024 | Chian | WQX:30  Control:30 | 32/28 | WQX:56.10  Control:55.25 | WQX  60min/day  4weeks | UPDRS-Ⅲ;HAMD;BBS;PDQ-39 |
| L Ding | 2021 | China | TC:40  Control:40 | 46/34 | TC:64.1  Control:66.9 | TC  75min/day  8weeks | BBS;TUGT |
| XZ Kong | 2022 | China | WQX:46  Control:46 | 54/38 | WQX:66.1  LIE:65.79 | WQX  4weeks | BBS |
| Y Zhu | 2021 | China | TC：41  Control：41 | 49/33 | TC：64.55  Control：64.52 | TC  60min/day  5day/week  8weeks | BBS |
| TY Jin | 2023 | China | TC1:30  TC2:30  Control:30 | 52/38 | TC1:70.77  TC2:64.69  Control:68.53 | TC1  30min/day  5day/week  12weeks  TC2  30min/day  5day/week  12weeks | BBS;TUGT;UPDRS-Ⅲ |
| L Liu | 2019 | Chian | BDJ：40  Control：40 | 46/34 | BDJ：55.61  Control：50.15 | BDJ  3weeks | HAMD |
| D Yang | 2019 | China | BDJ:50  Control:50 | 61/39 | BDJ:50  Control:50 | BDJ  8weeks | BBS |
| CJ Li | 2019 | China | BDJ:33  Control:33 | 39/27 | BDJ:62.88  Control:62.42 | BDJ  30min/day  4weeks | HAMD |
| Y You | 2020 | China | TC:35  Control:35 | 37/33 | TC:68.49  Control:68.81 | TC  60min/day  2day/week  24weeks | BBS;HAMD;UPDRS-Ⅲ |
| JZ Wang | 2016 | China | TC:40  Control:40 | 39/41 | TC:67.6  Control:68 | TC  50-60min/day  16weeks | BBS;HAMD;UPDRS-Ⅲ |
| HL Xiao | 2021 | China | TC:20  Control:20 | 17/23 | TC:72.78  Control:72.58 | TC  60min/day  4day/week  24weeks | BBS |
| WY Deng | 2020 | China | TC:50  Control:50 | 49/51 | TC:54.5  Control:54.4 | TC  60min/day  5day/week  12weeks | BBS |
| L Ding | 2023 | China | TC:42  Control:42 | 50/34 | TC:69.12  Control:69.34 | TC  60min/day  4day/week  12weeks | BBS |
| L Li | 2017 | China | TC:42  Control:38 | 44/36 | TC:65.25  Control:67.78 | TC  60min/day  3day/week  16weeks | UPDRS-Ⅲ |
| H Yang | 2023 | China | WQX:48  Control:47 | 68/27 | WQX:65.13  Control:64.89 | WQX  30-50min/day  5day/week  16weeks | UPDRS-Ⅲ |
| GJ Zhang | 2022 | China | WQX:60  Control:60 | 68/52 | 67.37 | WQX  45min/day  6weeks | BBS;HAMD;PDQ-39;UPDRS-Ⅲ |
| GQ Chen | 2016 | China | TC:15  Control:15 | 13/17 | TC:68.1  Control:68.2 | TC  50-60min/day  8weeks | BBS |

M/F:male/female;QG:qigong;LZJ:liuzijue;WQX:wuqinxi;YJJ:yijinjing;BDJ:baduanjin;TC:Tai Chi chuan;PDQ-39:Parkinson's disease questionnaire-39;BBS:Berg Balance Scale;TUGT:Timed 'Up and Go' Test;UPDRS-Ⅲ-III:Unified Parkinson’s Disease Rating Scale-Ⅲ;HAMD:Hamilton Depression Scale;RT:Resistance Training;SE: Stretching Exercise;BK: Brisk Kinetics

(1-59)

1. Schmitz-Hübsch T, Pyfer D, Kielwein K, Fimmers R, Klockgether T, Wüllner U. Qigong exercise for the symptoms of Parkinson's disease: a randomized, controlled pilot study. Movement disorders. 2006;21(4):543–8.

2. Hackney ME, Earhart GM. Tai Chi improves balance and mobility in people with Parkinson disease. Gait & posture. 2008;28(3):456–60.

3. Zhu Yi, Li Jianxing, Li Ning, Jin Hongzhu, Hua Liang, Dong Qing. The Effect of Tai Chi on Motor Control in Early Parkinson's Disease. 2011; 17(4).

4. Li F, Harmer P, Fitzgerald K, Eckstrom E, Stock R, Galver J, et al. Tai chi and postural stability in patients with Parkinson's disease. New England journal of medicine. 2012;366(6):511–9.

5. Amano S, Nocera JR, Vallabhajosula S, Juncos JL, Gregor RJ, Waddell DE, et al. The effect of Tai Chi exercise on gait initiation and gait performance in persons with Parkinson's disease. Parkinsonism & Related Disorders. 2013;19(11):955–60.

6. Nocera JR, Amano S, Vallabhajosula S, Hass CJ. Tai Chi Exercise to Improve Non-Motor Symptoms of Parkinson's Disease. Journal of yoga & physical therapy. 2013;3.

7. Gao Q, Leung A, Yang Y, Wei Q, Guan M, Jia C, et al. Effects of Tai Chi on balance and fall prevention in Parkinson's disease: a randomized controlled trial. Clin Rehabil. 2014;28(8):748–53.

8. Chen Guoqiang, Yang Pu, Huang Hao, Lu Juanjuan, Sun Hui, Deng Xiang, et al. The efficacy of exercise therapy combined with Tai Chi on patients with Parkinson's disease. 2016;31(6):459–60.

9. Guan Xihong, Tang Xiaozhu, Liu Jianmin. The influence of Tai Chi training on the walking ability and fear of falling in patients with Parkinson's disease. 2016;30(28):3514–7.

10. Ji Suqiong, Mao Zhijuan, Yang Qingmei, Gao Hongling, Xue Zheng. Observation on the efficacy of Tai Chi exercise on patients with Parkinson's disease. 2016;31(1):51–3.

11. Wang Jianzhong, Peng Youjing, Zheng Zhixiong. Research on the efficacy of Tai Chi for depression in early Parkinson's disease patients %J Medical Theory and Practice. 2016;29(19):3309–11.

12. Schmidt MA. Mood symptoms in Parkinson's disease and their impact on a Qigong exercise's efficacy for treating sleep quality and gait performance2016.

13. Xiao CM, Zhuang YC. Effect of health Baduanjin Qigong for mild to moderate Parkinson's disease. Geriatrics & gerontology international. 2016;16(8):911–9.

14. Li Lin, Yu Liying, Wang Dandan, Wan Xiaocheng. The Effects of Tai Chi Exercise on Gait and Posture Control in Patients with Mild to Moderate Parkinson's Disease. 2017; 39(7): 535–8.

15. Liu Xiaolei, Wan Zhirong, Shang Mengqing, Yang Liuxin, Xing Yan. Observation on the Therapeutic Effect of Fitness Qigong Exercise on Patients with Parkinson's Disease %J Chinese Journal of Neuroimmunology and Neurology. 2017; 24(01): 34–7.

16. Lu Fulin. The Impact of Tai Chi on the Quality of Life of Elderly Patients with Early Parkinson's Disease. 2017; 37(20): 5121–3.

17. Guan Xihong, Tang Xiazhu, Dong Yonghai. The Effects of Tai Chi Training on Walking Ability and Fear of Falling in Early Parkinson's Disease Patients. 2018; 38(20).

18. Wu Tingting, Wang Yaqun, Luo Xiangru, Ye Su. The Effects of Tai Chi Exercise on Cognitive Function and Health-Related Quality of Life of Patients with Parkinson's Disease. 2018; 33(2): 95–7.

19. Lee HJ, Kim SY, Chae Y, Kim MY, Yin C, Jung WS, et al. Turo (Qi Dance) Program for Parkinson's Disease Patients: randomized, Assessor Blind, Waiting-List Control, Partial Crossover Study. Explore (New York, NY). 2018;14(3):216–23.

20. Vergara-Diaz G, Osypiuk K, Hausdorff JM, Bonato P, Gow BJ, Miranda JG, et al. Tai Chi for Reducing Dual-task Gait Variability, a Potential Mediator of Fall Risk in Parkinson's Disease: a Pilot Randomized Controlled Trial. Global advances in health and medicine. 2018;7:2164956118775385.

21. Li Cuijing, Chang Hong, Wei Na, Qu Miao, Liu Fengchun. Observation on the intervention effect of Baduanjin exercise therapy on the quality of life and depressive symptoms of patients with Parkinson's disease and mild to moderate depression. 2019;38(11):1129–31.

22. Liu Li, Tang Yao, Wang Guangying. Observation on the nursing efficacy of traditional Chinese medicine characteristic nursing intervention for patients with Parkinson's disease and depressive state. 2019;35(05):61–2.

23. Yang Dan, Peng Xiaojing. Observation on the early rehabilitation effect of Baduanjin fitness exercise on patients with Parkinson's disease with balance disorder and analysis of nursing countermeasures. 2019(3):224.

24. Deng Wanying. Analysis of the clinical effect of Taijiquan training on improving the balance function and fear of falling in Parkinson's patients. 2020;10(17):28.

25. Wang Wanhong, Bi Hongyan, Qiu Zhenangang, Liu Xihua, Zhuang He, Hao Shijie, et al. The influence of visual tracking training combined with the Six Character Formula on the motor function and quality of life of Parkinson's disease patients. 2020;30(06):474–8.

26. You Hua, She Junhui. Group therapy of Taijiquan balance exercise in improving the balance function and depressive state of Parkinson's disease patients. 2020;44(07):1071–2.

27. Khuzema A, Brammatha A, Arul Selvan V. Effect of home-based Tai Chi, Yoga or conventional balance exercise on functional balance and mobility among persons with idiopathic Parkinson's disease: an experimental study. Hong kong physiotherapy journal. 2020;40(1):39–49.

28. Moon S, Sarmento CVM, Steinbacher M, Smirnova IV, Colgrove Y, Lai SM, et al. Can Qigong improve non-motor symptoms in people with Parkinson's disease - A pilot randomized controlled trial? Complement Ther Clin Pract. 2020;39:101169.

29. Zhu M, Zhang Y, Pan J, Fu C, Wang Y. Effect of simplified Tai Chi exercise on relieving symptoms of patients with mild to moderate Parkinson's disease. Journal of sports medicine and physical fitness. 2020;60(2):282–8.

30. Cao Haihao, Sun Wenyu, Xi Xiaoming, Wang Limin, Bi Hongyan. The Effects of Wuzhenxiu Qigong on Balance Function, Walking Ability and Quality of Life in Patients with Parkinson's Disease. 2021;27(09):1087–92.

31. Ding Li, Cheng Jianlan, Wan Xiaowei, Yu Xiaoming. Clinical Observation on the Balance Function and Walking Ability of Parkinson's Patients after Training of Taiji Yunshou Combined with Chinese Medicine Bath. 2021;42(09):96–8.

32. Han Wen, Dong Ting, Ding Xinyuan, Yun Zongjin. Application of Sensory Game Combined with Wuzhenxiu Qigong in Patients with Parkinson's Disease with Motor Disorders. 2021;43(9):1153–6, 60.

33. Shi Xinwei, Yang Weili, Ji Weijun. The Effects of Eightdanjing Combined with Balance Pad Training on Lower Limb Motor Function and Body Balance Ability of Elderly Patients with Parkinson's Disease. 2021;21(11).

34. Xiao Huiling, Hong Anhui, Ma Xiaozhen. Observation on the Efficacy of Taijiquan in Improving Balance Disorders in Early Parkinson's Disease Patients. 2021;12(8).

35. Zhu Yan, Zhao Songwei, Ding Zhenhuan, Shi Min. Clinical Observation of Xifeng Dingzhan Decoction Combined with Taijiquan in Treating Parkinson's Disease. Journal of Practical Traditional Chinese Medicine. 2021;37(2):206–8.

36. Shen M, Pi YL, Li Z, Song T, Jie K, Wang T, et al. The feasibility and positive effects of wuqinxi exercise on the cognitive and motor functions of patients with parkinson's disease: a pilot study. Evidence-based complementary and alternative medicine. 2021;2021.

37. Kong Xiangzhen, Kong Xixi, Hu Yue, Zhang Ning. Application of Five-Animal Play Combined with Low-Load Exercise in the Rehabilitation and Care of Parkinson's Disease Patients. 2022;31(1).

38. Zhang Guojuan, Zhuang Hongxia, Li Juan, Wu Qi, Huang Rong, Zhang Lili. Application of Five-Animal Play Combined with Physical Training in Patients with Motor Disorders of Parkinson's Disease. 2022;14(5):287–90.

39. Li G, Huang P, Cui SS, Tan YY, He YC, Shen X, et al. Mechanisms of motor symptom improvement by long-term Tai Chi training in Parkinson's disease patients. Translational neurodegeneration. 2022;11(1):6.

40. Li X, Taylor A, Li J, Wang T, Kuang J, Zhang Z, et al. Effects of Health Qigong Exercise on Depression and Anxiety in Patients with Parkinson?s Disease. International Journal of Mental Health Promotion. 2022;24(6):855–67.

41. Li Z, Wang T, Shen M, Song T, He J, Guo W, et al. Comparison of Wuqinxi Qigong with Stretching on Single-and Dual-Task Gait, Motor Symptoms and Quality of Life in Parkinson’s Disease: A Preliminary Randomized Control Study. International Journal of Environmental Research and Public Health. 2022;19(13).

42. Zhen W, Yanling P, Xiaoyin T, Chen R, Yu L, Wei G, et al. Effects of Wu Qin Xi exercise on reactive inhibition in Parkinson's disease: a randomized controlled clinical trial. Frontiers in aging neuroscience. 2022;14:1–13.

43. Ding Li, Cheng Jianlan, Yu Xiaoming, Wan Xiaowei, Xiong Jing. The Effects of Tai Chi Training on the Mental Health and Balance Ability of Parkinson's Disease Patients. 2023;23(3):19–21.

44. Jiang Jiahui, Bi Hongyan. The Impact of Qigong Yi Jin Jing on Fatigue and Quality of Life of Patients with Mild to Moderate Parkinson's Disease. 2023;37(24):4452–7.

45. Jin Tianyu, Cao Fangzheng, Zhang Zheyu, Jiang Bingze, Xu Liang'e, Xu Bin. The Effects of Moxibustion Combined with Tai Chi Yunshou Training on the Balance Function of Parkinson's Disease Patients. 2023;25(1):73–5.

46. Wang Meihua, Gan Min, Wu Haiqin, Yu Danxia, Chen Zhihua, Qian Ping'an, et al. The Effects of Different Training Loads of Tai Chi Exercise on the Rehabilitation Efficacy of Early and Mid-stage Parkinson's Disease Patients. 2023;50(10).

47. Yang Hui, Lou Xiran, Li Yanan. The Effects of Five-Animal Play Exercise on Patients with Parkinson's Disease and Motor Disorders. 2023;22(3).

48. Meng L, Wang D, Shi Y, Li Z, Zhang J, Lu H, et al. Enhanced brain functional connectivity and activation after 12-week Tai Chi-based action observation training in patients with Parkinson’s disease. Frontiers in aging neuroscience. 2023;15.

49. Chen Hua, Qin Zhenhua. Analysis of the Effect of Xueyu Qingxin Decoction Combined with Five-Animal Exercise on the Treatment of Parkinson's Disease. 2024;15(22).

50. He Limi, Chen Ting, Li Hong. Observation on the Efficacy of Eight-Section Exercises Combined with Anguo Music for Patients with Parkinson's Depression of Liver Qi Stagnation Type. 2024;19(14):128–31.

51. Lu Chunxiu, Chen Yuejing, Fu Haipeng, Dong Ku, Zeng Yiqi, Mo Xianrong. The Efficacy of Eight-Section Exercises Combined with Home-Based Parkinson Rehabilitation Exercises on Negative Emotions of Early and Mid-stage Parkinson's Disease Patients. 2024;5(5):64–7.

52. Lu Chunxiu, Zheng Youzhen, Mo Xianrong, Xie Shenghua, Dong Ku, Wang Chunyan. 30 Cases of Parkinson's Disease Patients with Motor Dysfunction Treated by Eight-Section Exercises Combined with G-EO Rehabilitation Robot. 2024;40(4):18–21.

53. Chang C-L, Pan CY, Wang T-C, Tseng YT, Chien C-Y, Lin T-K, et al. Distinct effects of long-term Tai Chi Chuan and aerobic exercise interventions on motor and neurocognitive performance in early-stage Parkinson's disease: a randomized controlled trial. European Journal of Physical and Rehabilitation Medicine. 2024;60(4):621–33.

54. Li KF, Li J, Xia AL, Wang XW, Wang AL, Shi Y, et al. The effects of Baduanjin on fine motor skills in mild and moderate Parkinson's disease: A randomized controlled trial. Clinical Parkinsonism and Related Disorders. 2024;11.

55. Zeng Liang, Gu You, Hu Jiawei, Yang Huilan, Dong Xiaowei, Pan Guoliang, et al. Clinical Observation on the Improvement of Motor Function in Early and Mid-stage Parkinson's Disease Patients by Acupuncture, Massage and Eight-Section Brocade Exercises. 2025;23(2):151–8.

56. Li Qian, Liu Huimiao, Wang Wenting, Zhang Sai, Xie Bingchuan, Chen Lei, et al. The Effect of Eight-Section Brocade Exercises on the Emotion and Sleep Quality of Patients with Mild to Moderate Parkinson's Disease. 2025;42(3).

57. Yang Min, Shi Dingling, Kuang Wei, Gao Xiaolin. Research on the Effects of Tai Chi on Motor Symptoms and Serum IL-1β in Early Parkinson's Patients. 2025;43(2).

58. Park J, Cheon S-M, Lee MJ, Ryu D-W, Yoo D. Comparison of the Impact of Various Exercise Modalities on Parkinson's Disease. Journal of Movement Disorders. 2025.

59. Yin H, Cheng O, Zhang X, Quan F, Zhang Y, Zuo H, et al. Effects of Liuzijue Qigong on respiratory function among patients with Parkinson's disease: a randomized clinical trial. BMC complementary medicine and therapies. 2025;25(1):63.

Table S3 Results of consistency modeling

| Outcomes | Consistency test | Inconsistency test | I2(%) |
| --- | --- | --- | --- |
| UPDRS-Ⅲ | 159.59 | 159.63 | 0 |
| BBS | 132.14 | 132.13 | 0 |
| PDQ-39 | 55.339 | 55.352 | 0 |
| TUGT | 89.173 | 89.083 | 5 |
| HAMD | 54.999 | 55.122 | 3 |

Table S4 League table of UPDRS-Ⅲ

| MD 95%CrI | | | | | | |
| --- | --- | --- | --- | --- | --- | --- |
| BDJ |  |  |  |  |  |  |
| -5.33 (-8.47, -2.17)^*^ | Control |  |  |  |  |  |
| -3.78 (-10.4, 2.75) | 1.56 (-4.23, 7.27) | LZJ |  |  |  |  |
| -1.14 (-5.72, 3.43) | 4.18 (0.86, 7.49)^*^ | 2.64 (-3.98, 9.27) | QG |  |  |  |
| -1.79 (-5.35, 1.77) | 3.54 (1.87, 5.19)^*^ | 1.98 (-3.98, 7.98) | -0.65 (-4.19, 2.89) | TC |  |  |
| -2.55 (-6.84, 1.87) | 2.79 (-0.16, 5.84) | 1.24 (-5.19, 7.81) | -1.4 (-5.79, 3.13) | -0.75 (-4.11, 2.73) | WQX |  |
| -3.08 (-10.55, 4.44) | 2.25 (-4.54, 9.05) | 0.7 (-8.15, 9.61) | -1.92 (-9.49, 5.63) | -1.29 (-8.27, 5.72) | -0.53 (-8, 6.8) | YJJ |

* Means P<0.05

Table S5 League table of BBS

| MD 95%CrI | | | | |
| --- | --- | --- | --- | --- |
| BDJ |  |  |  |  |
| 3.72 (1.82, 5.62)^*^ | Control |  |  |  |
| 2.63 (-2.57, 7.8) | -1.1 (-5.91, 3.72) | QG |  |  |
| -0.26 (-2.43, 1.92) | -3.98 (-5.02, -2.91)^*^ | -2.88 (-7.8, 2.07) | TC |  |
| 0.78 (-2.06, 3.6) | -2.94 (-5.04, -0.85)^*^ | -1.84 (-7.08, 3.39) | 1.04 (-1.31, 3.37) | WQX |

* Means P<0.05

Table S6 League table of TUGT

| MD 95%CrI | | | | | |
| --- | --- | --- | --- | --- | --- |
| BDJ |  |  |  |  |  |
| -3.56 (-7.06, -0.53)^*^ | Control |  |  |  |  |
| 3.22 (-2.86, 8.97) | 6.8 (1.87, 11.74)^*^ | LZJ |  |  |  |
| -0.55 (-5.16, 3.73) | 3.01 (0.05, 6.12)^*^ | -3.78 (-9.5, 2.03) | QG |  |  |
| -1.91 (-5.54, 1.3) | 1.65 (0.61, 2.75)^*^ | -5.14 (-10.17, -0.1)^*^ | -1.36 (-4.63, 1.81) | TC |  |
| -0.08 (-4.08, 3.59) | 3.49 (1.44, 5.67)^*^ | -3.31 (-8.64, 2.1) | 0.46 (-3.22, 4.18) | 1.83 (-0.48, 4.23) | WQX |

* Means P<0.05

Table S7 League table of PDQ-39

| MD 95%CrI | | | | | | |
| --- | --- | --- | --- | --- | --- | --- |
| BDJ |  |  |  |  |  |  |
| -11.92 (-24.65, 0.69) | Control |  |  |  |  |  |
| -10.01 (-25.01, 4.81) | 1.91 (-5.87, 9.83) | LZJ |  |  |  |  |
| -19.61 (-39.24, 0.41) | -7.59 (-22.88, 7.77) | -9.51 (-26.72, 7.63) | QG |  |  |  |
| -8.48 (-21.97, 4.88) | 3.44 (-0.98, 7.88) | 1.54 (-7.52, 10.46) | 11.05 (-4.9, 27.04) | TC |  |  |
| -6.45 (-19.52, 6.52) | 5.44 (2.45, 8.6)^*^ | 3.55 (-4.89, 11.92) | 13.06 (-2.56, 28.69) | 2 (-3.32, 7.44) | WQX |  |
| 6.23 (-7.99, 20.24) | 18.14 (11.76, 24.49)^*^ | 16.25 (6.03, 26.21)^*^ | 25.72 (9.16, 42.26)^*^ | 14.71 (6.9, 22.43)^*^ | 12.71 (5.48, 19.68)^*^ | YJJ |

* Means P<0.05

Table S8 League table of HAMD

| MD 95%CrI | | | | |
| --- | --- | --- | --- | --- |
| BDJ |  |  |  |  |
| -3.87 (-7.58, -0.21)^*^ | Control |  |  |  |
| -3.23 (-12.68, 6.25) | 0.63 (-8.01, 9.4) | LZJ |  |  |
| 1.33 (-4.03, 6.52) | 5.19 (1.36, 8.93)^*^ | 4.55 (-5.02, 13.95) | TC |  |
| -1.45 (-7.43, 4.48) | 2.41 (-2.26, 7.1) | 1.78 (-8.17, 11.64) | -2.78 (-8.71, 3.31) | WQX |

* Means P<0.05

Table S9 Results of Heterogeneity Test

| Outcomes | Pairwise meta-analysis | No of study | Heterogeneity (%) | MD 95%CrI |
| --- | --- | --- | --- | --- |
| HAMD | Control vs BDJ | 5 | 96.6 | 3.88（0.21,7.58） |
|  | LZJ vs Control | 1 | NA | -0.6（-9.43,8.27） |
|  | TC vs Control | 5 | 96 | -5.20（-8.92,-1.29） |
|  | WQX vs Control | 3 | 0 | -2.41(-7.12,2.3) |
| Outcomes | Pairwise meta-analysis | No of study | Heterogeneity (%) | MD 95%CrI |
| TUGT | Control vs BDJ | 2 | 92.8 | 3.59(0.53,7.04) |
|  | LZJ vs Control | 1 | NA | -6.80(-11.69,-1.80) |
|  | QG vs Control | 2 | 59.7 | -3.02(-6.18,-0.05) |
|  | TC vs Control | 14 | 87.5 | -1.65(-2.76,-0.59) |
|  | WQX vs Control | 4 | 71 | -3.51(-5.71,-1.46) |
| Outcomes | Pairwise meta-analysis | No of study | Heterogeneity (%) | MD 95%CrI |
| BBS | Control vs BDJ | 6 | 87.8 | -3.71(-5.62,-1.81) |
|  | QG vs Control | 1 | NA | 1.12(-3.72,5.94) |
|  | TC vs Control | 15 | 98.2 | 3.98(2.92,5.02) |
|  | WQX vs Control | 5 | 65.8 | 2.94(0.85,5.05) |
| Outcomes | Pairwise meta-analysis | No of study | Heterogeneity (%) | MD 95%CrI |
| PDQ-39 | Control vs BDJ | 1 | NA | 12.02(-0.78,24.73) |
|  | LZJ vs Control | 2 | 0 | -1.87(-9.76,5.90) |
|  | QG vs Control | 2 | 25.3 | 7.97(-7.16,22.84) |
|  | TC vs Control | 4 | 0 | -3.38(-7.83,1.03) |
|  | WQX vs Control | 6 | 69.7 | -5.45(-8.58,-2.44) |
|  | YJJ vs Control | 1 | NA | -18.1(-24.4,-11.7) |
| Outcomes | Pairwise meta-analysis | No of study | Heterogeneity (%) | MD 95%CrI |
| UPDRS-Ⅲ | Control vs BDJ | 6 | 93.4 | 5.33(2.09,8.52) |
|  | LZJ vs Control | 2 | 0 | -1.55(-7.41,4.34) |
|  | QG vs Control | 6 | 82.2 | -4.03(-7.77,-0.24) |
|  | TC vs Control | 21 | 85.2 | -3.57(-5.29,-1.85) |
|  | WQX vs Control | 6 | 60.4 | -2.82(-5.88,0.22) |
|  | YJJ vs Control | 1 | NA | -2.28(-9.15,4.67) |
|  | TC vs QG | 1 | NA | 1.29(-5.95,8.60) |

Table S10 GRADE Summary of Findings

| Outcome | Comparison | Effect size | Certainty of evidence | Risk of bias | Inconsistency | Indirectness | Imprecision | Publication bias | Downgrade reason |
| --- | --- | --- | --- | --- | --- | --- | --- | --- | --- |
| UPDRS-III | Baduanjin vs Control | MD -5.33 | ⊕⊕⊕◯ Moderate | Serious | Not serious | Not serious | Not serious | Not detected | Downgraded 1 level (risk of bias) |
| UPDRS-III | Tai Chi vs Control | MD -3.54 | ⊕⊕⊕◯ Moderate | Serious | Not serious | Not serious | Not serious | Not detected | Downgraded 1 level (risk of bias) |
| UPDRS-III | Qigong vs Control | MD -4.18 | ⊕⊕◯◯ Low | Serious | Not serious | Not serious | Serious | Not detected | Downgraded 2 levels (RoB + imprecision) |
| BBS | Tai Chi vs Control | MD 3.98 | ⊕⊕⊕◯ Moderate | Serious | Not serious | Not serious | Not serious | Not detected | Risk of bias |
| BBS | Baduanjin vs Control | MD 3.72 | ⊕⊕⊕◯ Moderate | Serious | Not serious | Not serious | Not serious | Not detected | Risk of bias |
| TUGT | Liuzijue vs Control | MD -6.80 | ⊕⊕◯◯ Low | Serious | Not serious | Not serious | Serious | Not detected | RoB + imprecision |
| PDQ-39 | Yijinjing vs Control | MD -18.14 | ⊕⊕⊕◯ Moderate | Serious | Not serious | Not serious | Not serious | Not detected | Risk of bias |
| HAMD | Tai Chi vs Control | MD -5.19 | ⊕⊕◯◯ Low | Serious | Not serious | Not serious | Serious | Not detected | RoB + imprecision |

Table S11 subgroup analysis results

| Outcome | Subgroup factor | Level | Effect size (MD) | 95% CI |
| --- | --- | --- | --- | --- |
| UPDRS-III | Duration | ≤12 weeks | -2.85 | (-4.90, -0.80) |
| UPDRS-III | Duration | >12 weeks | -4.92 | (-6.30, -3.54) |
| UPDRS-III | Frequency | ≤3/week | -3.1 | (-5.20, -1.00) |
| UPDRS-III | Frequency | >3/week | -5.01 | (-6.80, -3.22) |
| UPDRS-III | H&Y stage | 1–2 | -5.2 | (-7.10, -3.30) |
| UPDRS-III | H&Y stage | 3–5 | -2.4 | (-4.60, -0.20) |
| BBS | Duration | ≤12 weeks | 2.1 | (0.80, 3.40) |
| BBS | Duration | >12 weeks | 3.65 | (2.10, 5.20) |
| BBS | Frequency | ≤3/week | 2.45 | (0.90, 4.00) |
| BBS | Frequency | >3/week | 3.9 | (2.30, 5.50) |
| TUGT | Duration | ≤12 weeks | -2.3 | (-4.10, -0.50) |
| TUGT | Duration | >12 weeks | -4.9 | (-6.70, -3.10) |
| TUGT | Frequency | ≤3/week | -2.8 | (-4.50, -1.10) |
| TUGT | Frequency | >3/week | -4.6 | (-6.20, -3.00) |
| PDQ-39 | Duration | ≤12 weeks | -3.5 | (-6.20, -0.80) |
| PDQ-39 | Duration | >12 weeks | -6.8 | (-9.40, -4.20) |
| PDQ-39 | Dose | Low | -2.9 | (-5.60, -0.20) |
| PDQ-39 | Dose | High | -7.1 | (-10.20, -4.00) |
| HAMD | H&Y stage | 1–2 | -4.8 | (-7.10, -2.50) |
| HAMD | H&Y stage | 3–5 | -2.1 | (-4.30, 0.10) |


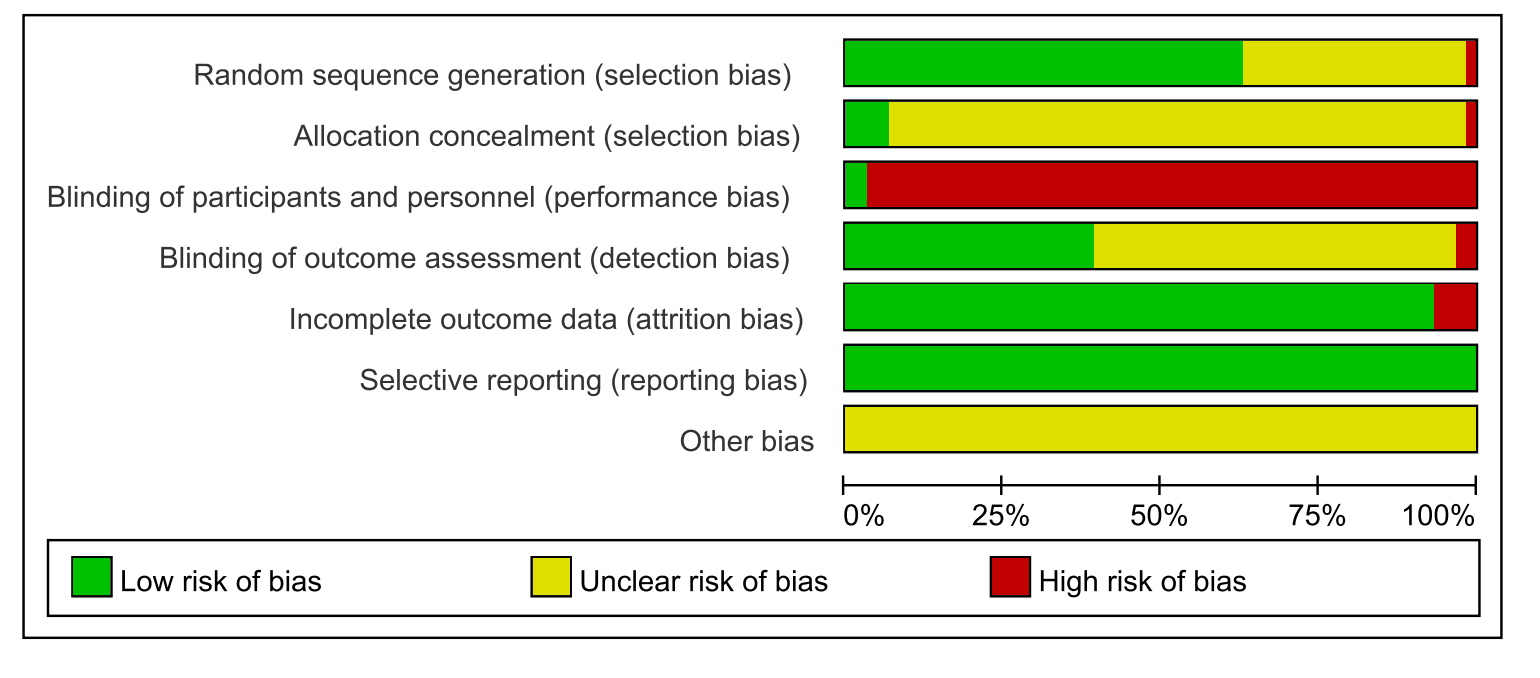
Figure S1 risk bias of graph


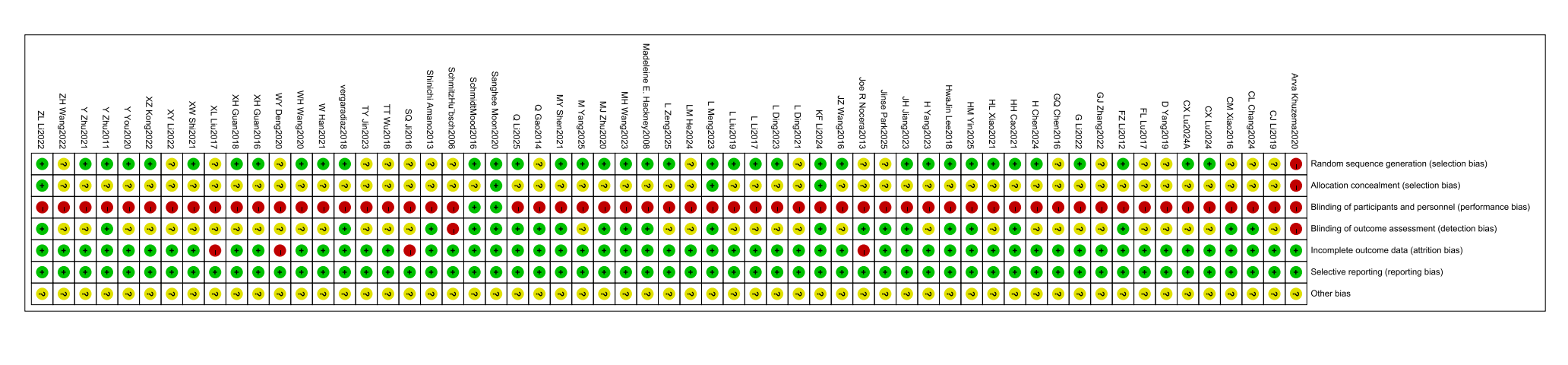
Figure S2 risk bias of summary


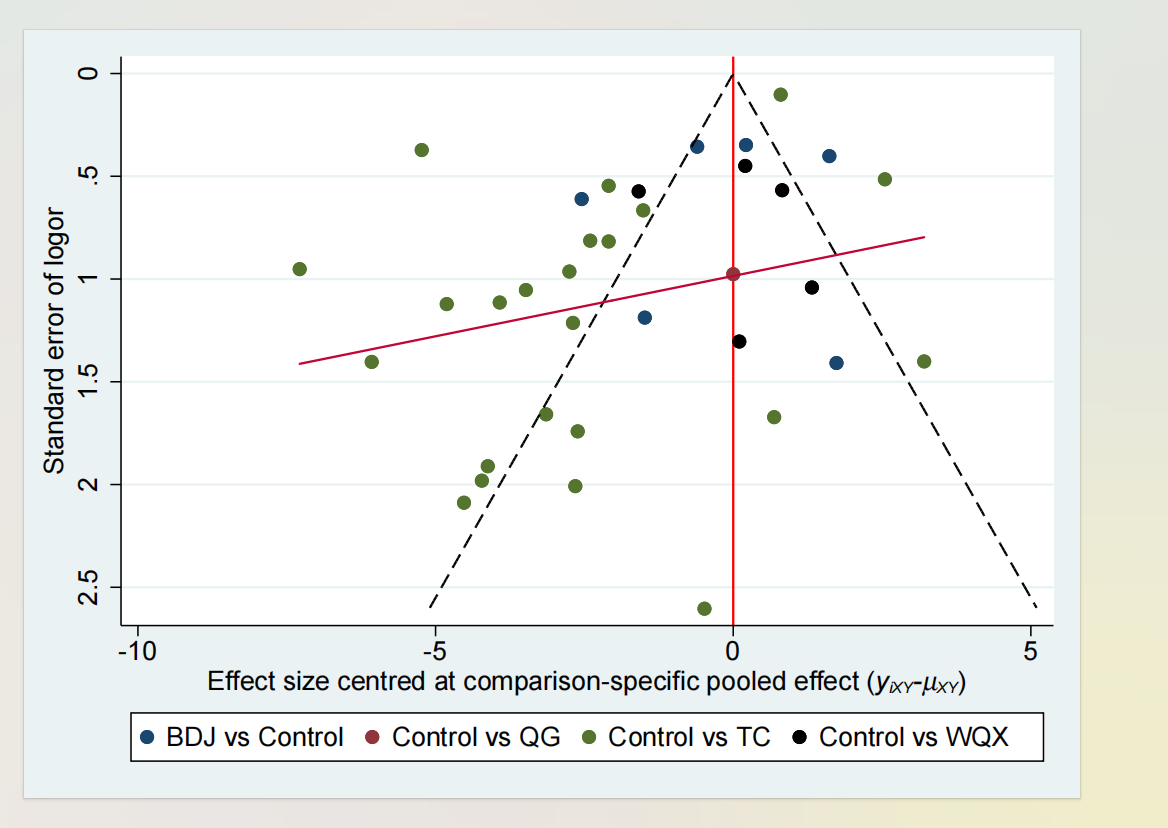


Figure S3 Funnel plot of the network meta-analysis of BBS


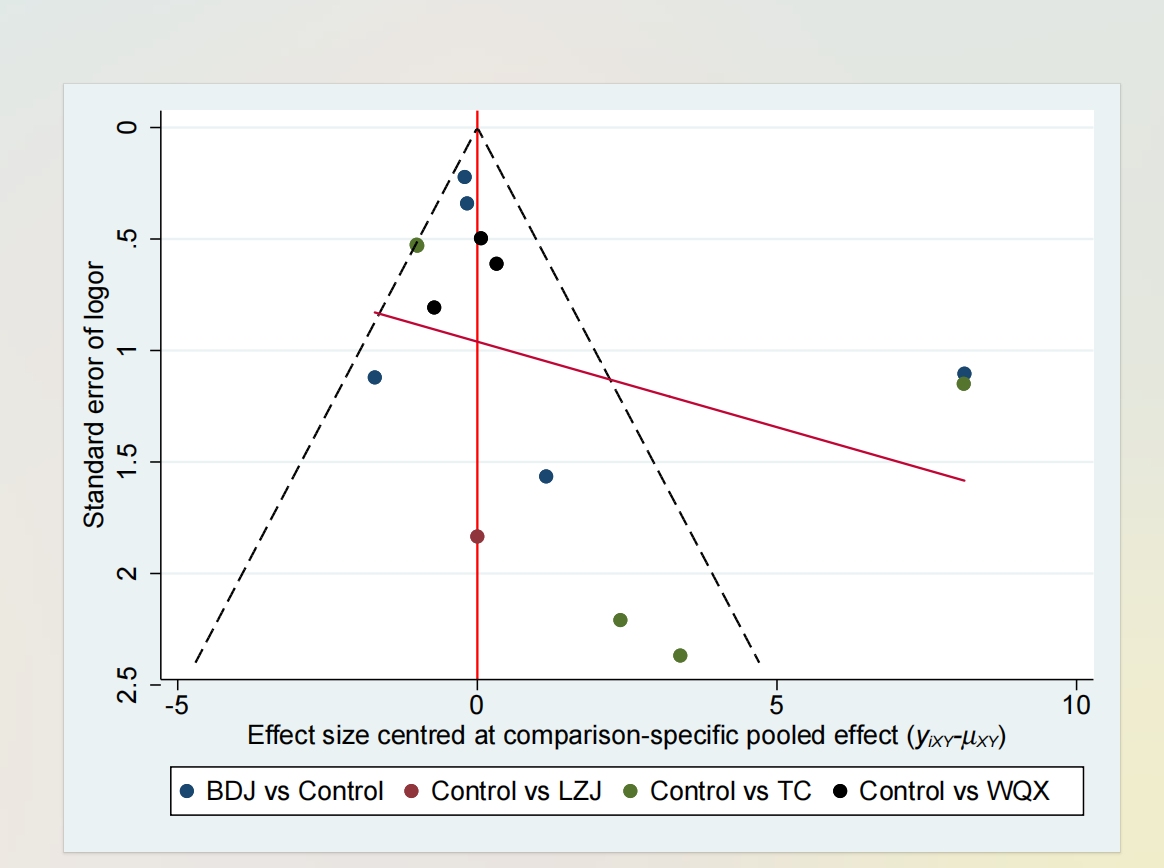


Figure S4 Funnel plot of the network meta-analysis of HAMD


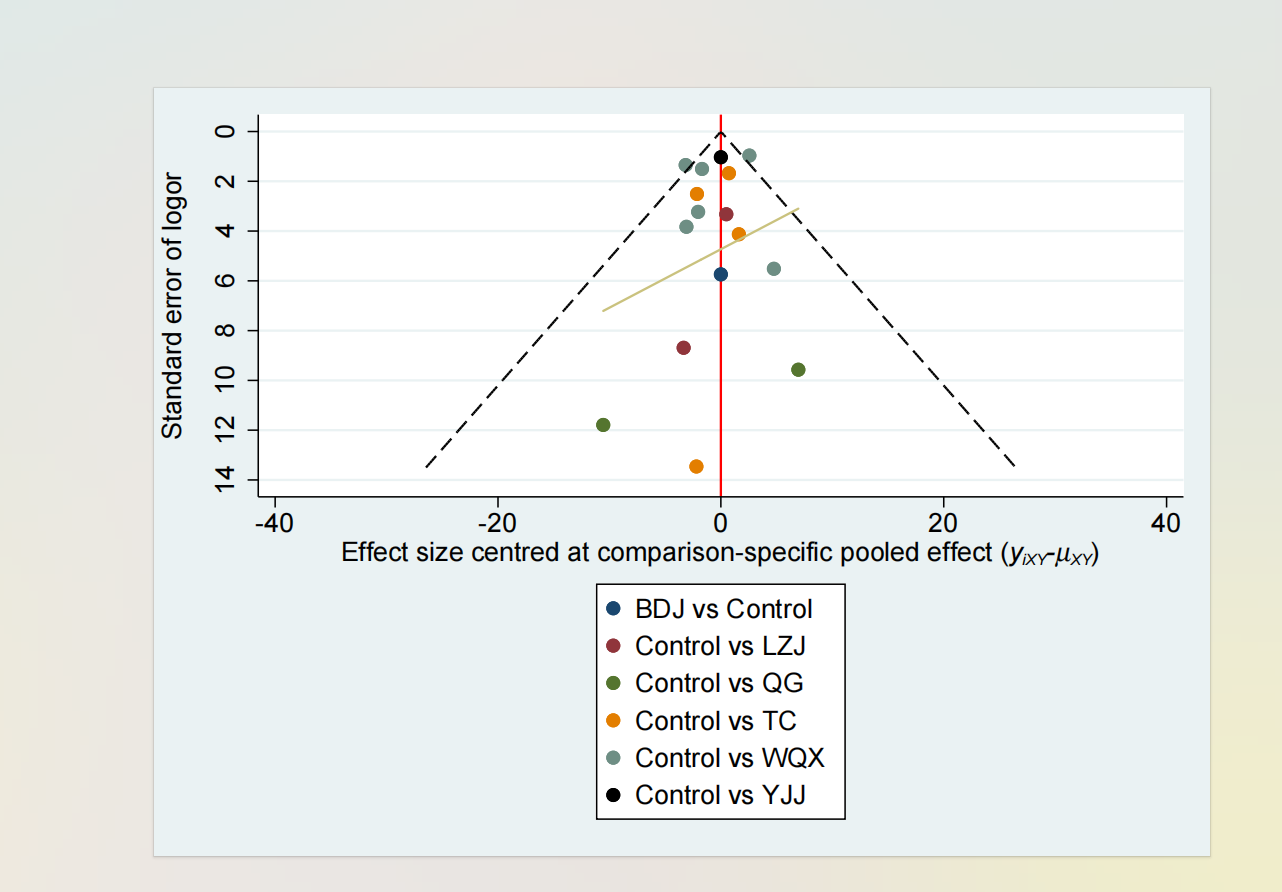


Figure S5 Funnel plot of the network meta-analysis of PDQ-39


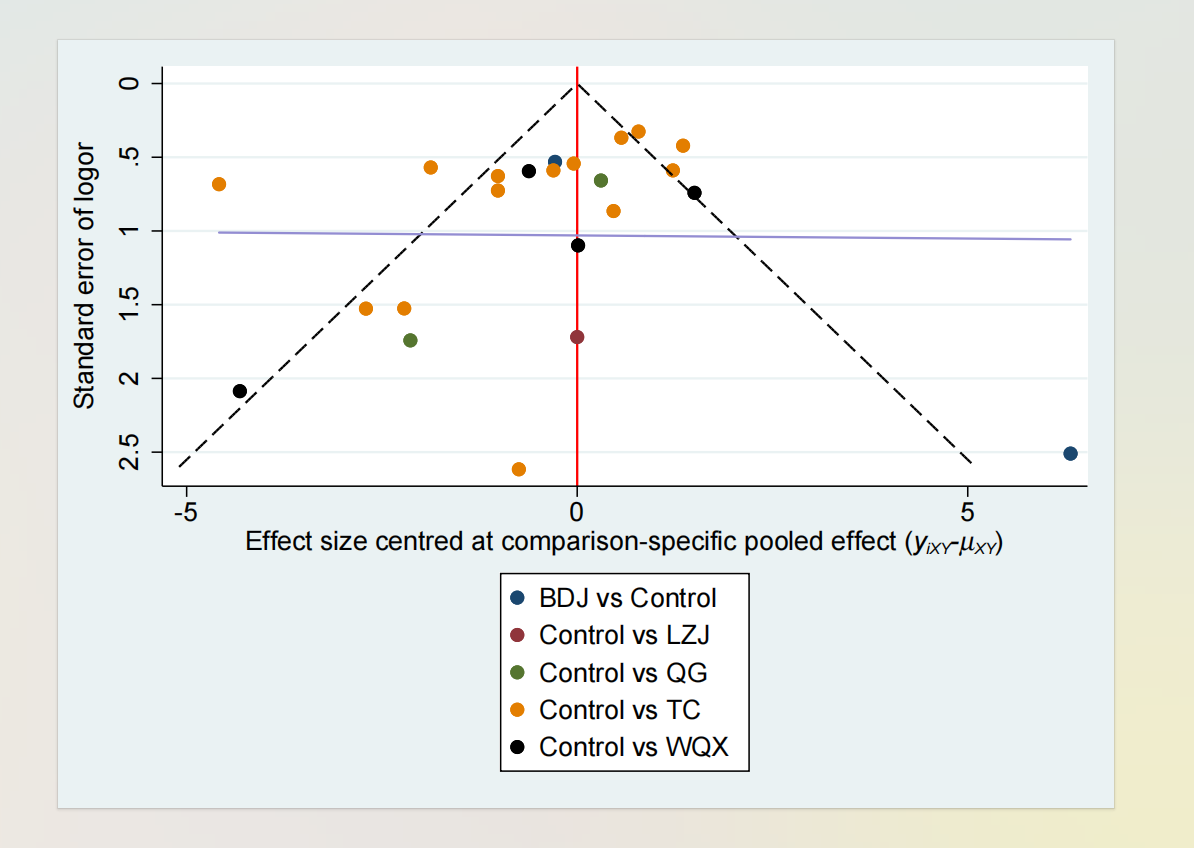


Figure S6 Funnel plot of the network meta-analysis of TUGT


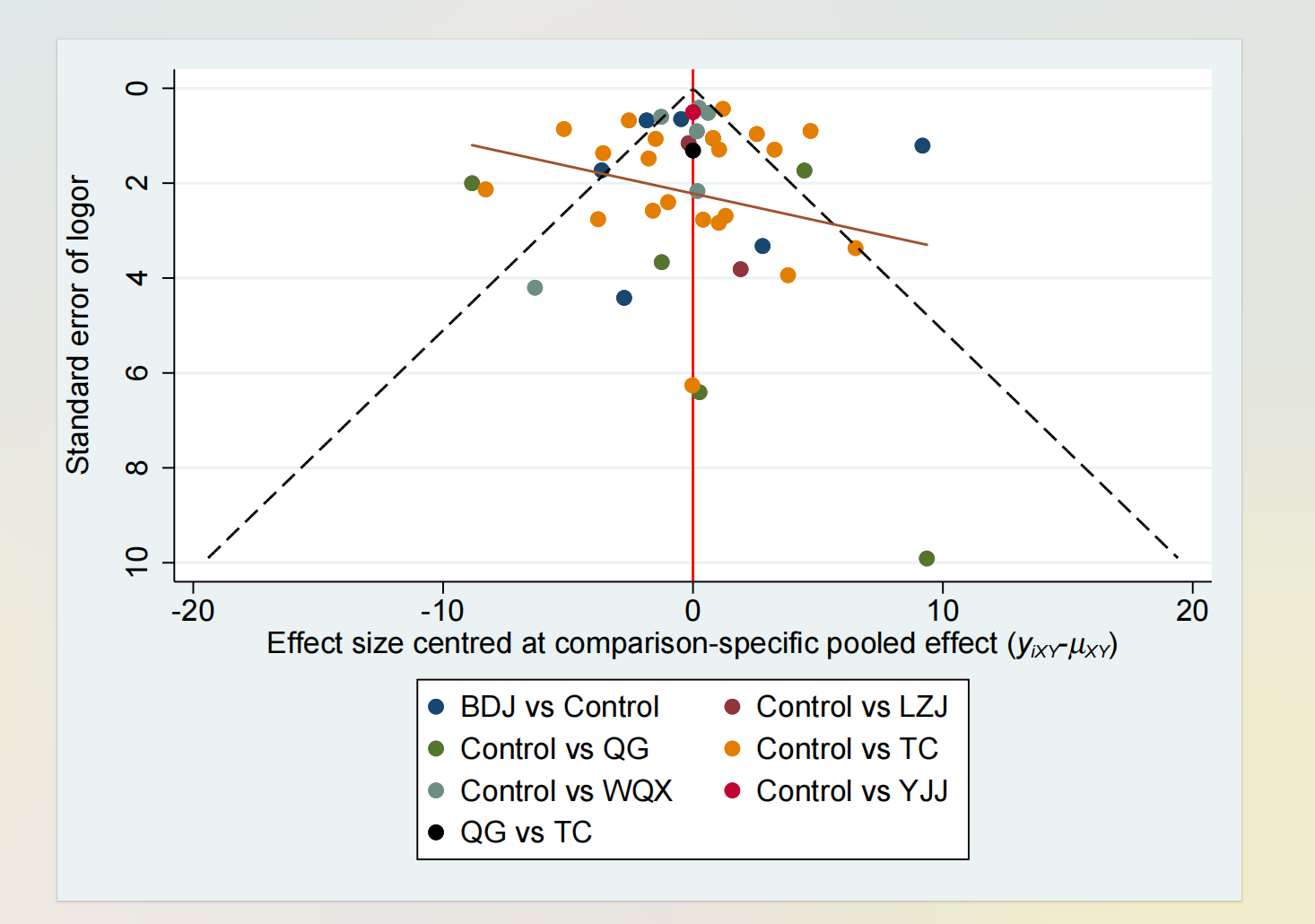


Figure S7 Funnel plot of the network meta-analysis of UPDRS-Ⅲ
