# Supplementary material for: Comparative efficacy of traditional Chinese medicine qigong exercise on motor and non-motor outcomes in Parkinson's disease: a network meta-analysis
Source: Front Neurol. 2026 Jul 15;17:1836123. doi: 10.3389/fneur.2026.1836123 (PMC13417681; doi:10.3389/fneur.2026.1836123)
Supplement: Supplementary file 2 [file Table_1.docx]

| Treatment | UPDRS-Ⅲ(%) | BBS(%) | PDQ-39（%） | TUGT（%） | HAMD (%) |
| --- | --- | --- | --- | --- | --- |
| BDJ | 84.7 | 74.1 | 79.6 | 62.6 | 68.6 |
| Control | 9.71 | 8.1 | 20.5 | 0.8 | 15 |
| LZJ | 34.8 | NR | 36.7 | 92.5 | 32.7 |
| QG | 69.4 | 29.8 | 7.8 | 53.5 | NR |
| TC | 59.6 | 82.2 | 46.7 | 27.7 | 84.4 |
| WQX | 48.2 | 55.8 | 61.9 | 62.8 | 49.3 |
| YJJ | 43.6 | NR | 96.7 | NR | NR |
